# Causal effects of gut microbiome on autoimmune liver disease: a two-sample Mendelian randomization study

**DOI:** 10.1186/s12920-023-01670-0

**Published:** 2023-10-03

**Authors:** Yugang Fu, Jiacheng Li, Yingying Zhu, Chong Chen, Jing Liu, Simin Gu, Yiyuan Zheng, Yong Li

**Affiliations:** 1 Department of Gastroenterology, Shanghai Municipal Hospital of Traditional Chinese Medicine, Shanghai University of Traditional Chinese Medicine, Zhijiang Middle Road 274#, Shanghai, Jing’an District China; 2 Municipal Medical College of Traditional Chinese Medicine, Shanghai University of Traditional Chinese Medicine, Shanghai, 200071 China

**Keywords:** Gut microbiome, Autoimmune liver disease, Mendelian randomization

## Abstract

**Background:**

Epidemiological studies have indicated a potential link between the gut microbiome and autoimmune liver disease (AILD) such as autoimmune hepatitis (AIH), primary biliary cholangitis (PBC), and primary sclerosing cholangitis (PSC). The relationship between the gut microbiome and autoimmune liver disease is still uncertain due to confounding variables. In our study, we aim to shed light on this relationship by employing a two-sample Mendelian randomization approach.

**Methods:**

We conducted a two-sample Mendelian randomization (MR) study using the R package "TwoSampleMR". The exposure data consisted of genetic variants associated with 194 bacterial traits obtained from the MiBioGen consortium. Summary statistics for AILD were obtained from the GWAS Catalog website. Furthermore, a series of sensitivity analyses were performed to validate the initial MR results.

**Results:**

There were two, four and three bacteria traits associated with an increased risk of AIH. PBC, and PSC respectively. In contrast, there were five, two and five bacteria traits associated with a decreased risk for AIH, PBC and PSC. Notably, the *genus_Clostridium_innocuum_group* showed a negative association with AIH (OR = 0.67, 95% CI: 0.49–0.93), and the *genus_Actinomyces* was found to be genetically associated with a decreased risk of PSC (OR = 0.62, 95% CI: 0.42–0.90).

**Conclusions:**

Our study identified the causal impact of specific bacterial features on the risk of AILD subtypes. Particularly, the *genus_Clostridium_innocuum_group* and the *genus_Actinomyces* demonstrated significant protective effects against AIH and PSC respectively. These findings provide further support for the potential use of targeted probiotics in the management of AILD.

**Supplementary Information:**

The online version contains supplementary material available at 10.1186/s12920-023-01670-0.

## Background

Autoimmune liver disease (AILD) is a rare chronic liver disorder characterized by autoimmune abnormalities. It encompasses three main types: autoimmune hepatitis (AIH), primary biliary cholangitis (PBC), and primary sclerosing cholangitis (PSC). The diagnosis of AILD typically involves a combination of specific autoantibody testing, serum biochemistry, and liver histology [1, 2]. Notably, specific autoantibodies associated with PSC have not been identified, and non-invasive imaging techniques are recommended for evaluating liver and bile duct fibrosis [3]. Despite the low incidence and prevalence of autoimmune liver diseases, they impose a disproportionate clinical burden on affected individuals. The global incidence rates per 100,000 population vary, ranging from 0.4 to 2.39 for AIH, 0.84 to 2.75 for PBC, and 0.1 to 4.39 for PSC [4]. Treatment typically involves the use of immunosuppressants, which are generally effective but often require long-term administration, raising concerns regarding potential side effects and patient adherence to therapy [5].

The gut microbiota, considered a virtual metabolic organ, is increasingly recognized for its role in various extraintestinal systems [6]. Recent research has emphasized the importance of the gut-liver axis in liver disease pathogenesis, involving intestinal barrier homeostasis, bile-mediated liver communication, and the composition and function of the gut microbiota [7]. While the crucial involvement of the gut microbiota in alcohol-associated liver disease and non-alcoholic fatty liver disease has been extensively investigated, studies focusing on the relationship between AILD and the gut microbiota are limited [8]. Over the past decades, Genome-Wide Association Study (GWAS) has successfully identified genetic factors associated with AIH, PBC, and PSC [9]. These GWAS data can be repurposed to explore the causal relationship between the gut microbiota and AILD. Mendelian randomization (MR), a statistical method, offers an opportunity to infer causal effects by using single nucleotide polymorphisms (SNPs) as instrumental variables (IVs) [10]. Through MR analysis, the causal relationship between the gut microbiome and the occurrence of AIH, PBC, and PSC can be explored, providing valuable insights for clinical practice. Additionally, the results of MR analysis may also support the potential development of probiotic therapies targeting the gut microbiota for AILD.

## Methods

### Study design

**Fig. 1:**
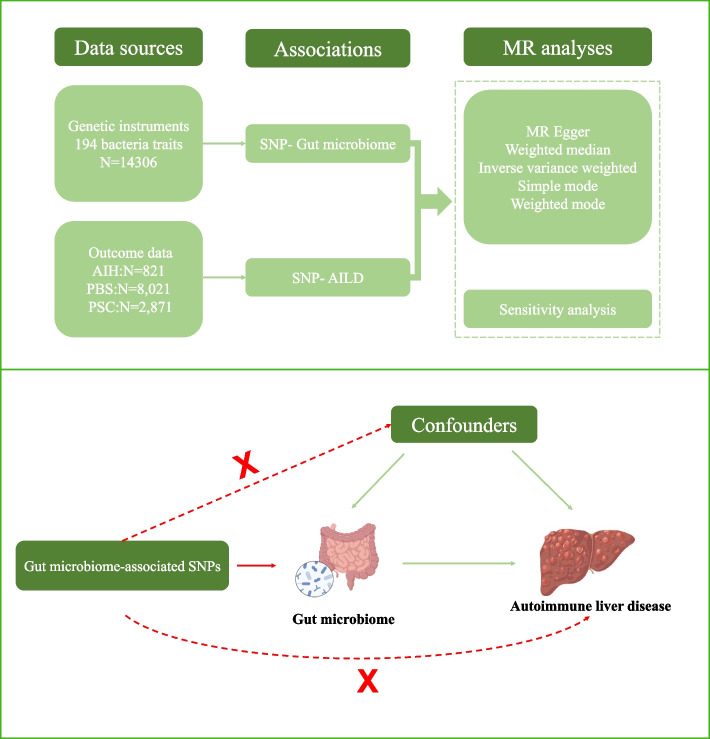
Study design of the two-sample Mendelian randomization for the effect of genetically predicted gut microbiome on AILD subtypes. AIH, autoimmune hepatitis; PBC, primary biliary cholangitis; PSC, primary sclerosing cholangitis; N: number of discovery cases; SNPs, single nucleotide polymorphisms

We conducted a two-sample MR study to investigate the causal association between the gut microbiome and autoimmune liver disease, and a workflow of the study is shown in Fig. 1. To ensure valid instrumental variables (IVs), three fundamental assumptions of the MR design were satisfied: (I) genetic variation as an IV must be significantly associated with the gut microbiome; (ii) genetic variation must be independent of confounders; and (iii) variation must be associated with autoimmune liver disease only through the gut microbiome [11]. The summary data were primarily based on independent GWAS and MR utilized SNPs to assess causality.

### GWAS data source

The full GWAS summary statistics for the microbiota were primarily derived from a large-scale multi-ethnic GWAS meta-analysis conducted by the international MiBioGen consortium [12], which was established to study the influence of human genetics on the gut microbiome. This meta-analysis included 211 gut microbiota and 122,110 related SNPs. To exclude the influence of ethnicity, GWAS data for participants of European ancestry were selected.

The summary statistics data for AIH, PBC, and PSC were acquired from the corresponding studies in the GWAS Catalog. For AIH (GCST90018785), there were 821 cases and 484,413 controls of European ancestry [13]. For PBC (GCST90061440), there were 8,021 cases and 16,489 controls of European ancestry [14]. For PSC (GCST004030), there were 2,871 cases and 12,019 controls of European ancestry [15]. Detailed descriptions of the study procedures, ethical approvals, and consent to participate can be found in the original studies.

### The selection of instrumental variables

To ensure the reliability of the results, instrumental variables were carefully selected. First, 17 bacterial traits with unknown classifications were excluded. Then SNPs with a *p*-value less than 10^–5^ were chosen, while those with weak associations or low minor allele frequencies were excluded. Independent SNPs were identified by clumping SNPs based on the European 1000 Genomes Project reference panel (r2 < 0.01 and clump distance > 10,000 kb). The instrumental strength of each SNP was assessed using the F statistics = (β/SE)^2^, and variables with F statistics values > 10 were excluded [16]. The corresponding data of the selected SNPs was extracted from the GWAS outcome data, and proxy SNPs were not allowed. The selection process was carried out using the “TwoSampleMR” R package (version 0.5.6) [17]. It's worth noting that due to the absence of the rsID column in the GWAS summary data of AIH, a set of unique identifiers for distinguishing similar SNPs was unavailable. To address this, we utilized the SNP Annotation Tool [18] to derive rsID information based on the available chromosome number and position details. As the GWAS summary data were annotated in the GRCh37 version, we ensured the use of the same version for consistency during the querying process. This enabled us to obtain the necessary rsID information for the SNPs. This information was then matched with the outcome data and processed using the “format_data” function in the “TwoSampleMR” package to obtain standardized data. During the harmonizing process, positive strand alleles were inferred using allele frequencies for palindromes to ensure that the effects of SNPs on exposure corresponded to the same allele as the effects of SNPs on the outcome. Finally, as a quality control measure, exposure traits with less than 3 SNPs were excluded.

### Conduction of MR analyses

MR analyses were performed using inverse variance-weighted (IVW), weighted median, MR–Egger, and maximum likelihood methods to identify gut microbiome related to three subtypes of autoimmune liver diseases. IVW was used as the primary method assuming all SNPs are valid variables. The weighted median approach yields consistent estimates assuming more than half of the weights are from valid SNPs [19]. MR–Egger analysis can calibrate for pleiotropy and calculate causal inferences even when all genetic variants are pleiotropic [20]. The maximum likelihood-based approach can generate appropriate confidence interval (CI) estimation when weak IV is observed. Guidance on interpreting the outcomes of these methods can be found elsewhere [21]. We further visualized the results of IVW method with a heatmap for more intuitive interpretation. Finally, several essential sensitivity analyses were performed to verify the robustness of the MR analysis results. A test for heterogeneity was conducted using Cochran’s test. MR-Pleiotropy Residual Sum and Outlier (MR-PRESSO) was performed to examine horizontal pleiotropy if available in order to eliminate SNPs with horizontal pleiotropic outliers [22]. The MR–Egger regression intercept was used to estimate potential pleiotropy of SNP, with a *P*-value > 0.05 indicating no horizontal pleiotropy. A leave-one-out analysis was also used to detect pleiotropy caused by each SNP. Sensitivity analyses were conducted using the “TwoSampleMR” R package. Results were presented as odds ratios (OR) with respective 95% CI. *P*-values were two-sided and statistical significance was set at the 5% level.

## Results

### Instrument variables for gut microbiome

SNPs from 194 bacterial traits containing five biological levels (i.e. phylum, class, order, family, and genus) were included in our study. Detailed information (i.e. effect allele, other allele, beta, standard error, *p*-value, and F statistics) of the final SNPs for each bacterial trait is shown in Supplementary Table 1.

### Causal effects of the gut microbiome on autoimmune liver diseases

**Fig. 2:**
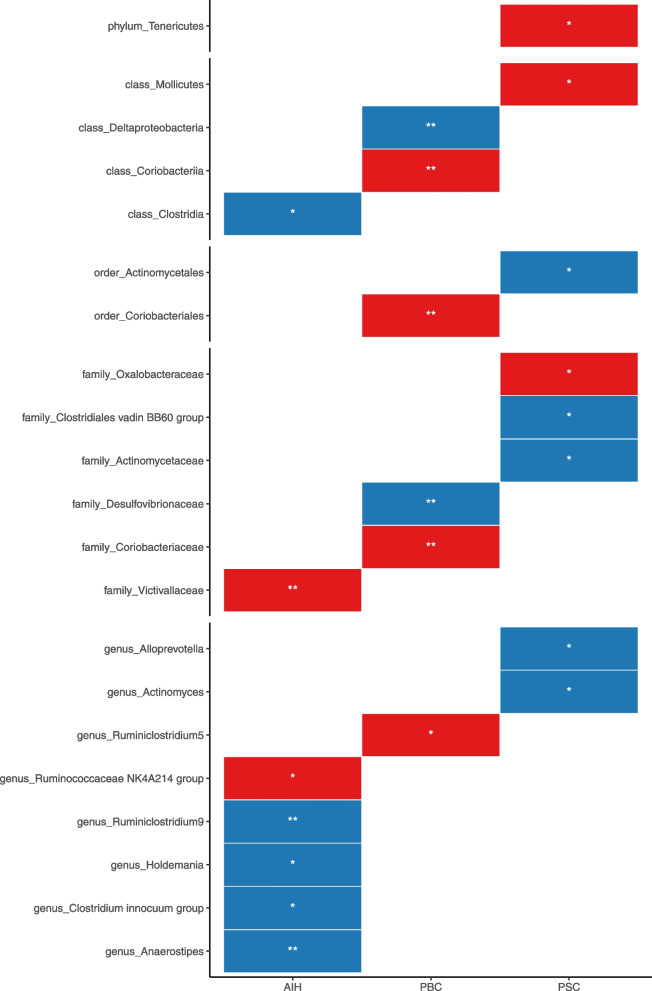
Heatmap illustrating positive IVW-MR results, with red indicating risk factors and blue indicating protective factors. Asterisks (*) denote statistical significance, with * indicating *P* < 0.05 and ** indicating *P* < 0.01

Using the IVW method as the primary MR detection method, a total of 21 gut microbiome traits were found to have a potential causal relationship with AILD (7 traits for AIH, 6 traits for PBC, and 8 traits for PSC). A heatmap was created to display these results, with red representing risk factors and blue representing protective factors (Fig. 2). These taxonomic groups demonstrate the hierarchical relationship between phyla, classes, orders, families, and genera of bacteria, with groups related at the highest level (phylum) and branching into more specific categories. In detail, the phylum *Tenericutes* includes the class *Mollicutes*. The class *Clostridia* contains two families: *Clostridiales vadin BB60 group* and *Victivallaceae*. The class *Coriobacteriia* includes the order *Coriobacteriales* and the family *Coriobacteriaceae*. The order *Actinomycetales* contains the family *Actinomycetaceae* and the genus *Actinomyces*. Genera in the results do not have a hierarchical relationship with each other. These results provide insight into the potential associations between certain bacterial features and the risk of AILD.

### AIH

According to our IVW-MR analysis, we found that genetically predicted levels of two bacterial features (*family_Victivallaceae*: OR 1.42, 95% CI 1.03–1.92, *P* = 0.004; *genus_Ruminococcaceae NK4A214 group*: OR 1.64, 95% CI 1.03–2.64, *P* = 0.039) were potentially associated with an decreased risk of AIH. Additionally, we observed that genetically predicted levels of five bacterial features were associated with a lower risk of AIH: *class_Clostridia* (OR 0.58, 95% CI 0.35–0.97, *P* = 0.039), *genus_Ruminiclostridium9* (OR 0.39, 95% CI 0.20–0.76, *P* = 0.005), *genus_Holdemania* (OR 0.66, 95% CI 0.46 to 0.95, *P* = 0.024). *genus_Clostridium innocuum group* (OR 0.67, 95% CI 0.49 to 0.93, *P* = 0.016) and *genus_Anaerostipes* (OR 0.35, 95% CI 0.17 to 0.74, *P* = 0.006) (Table 1). The aggressive role of *family_Victivallaceae* and the protectiove role of *genus_Anaerostipes* were supported by the Weighted Median method, whilst others were not (see Supplementary Table 2).

**Table 1:** Positive IVW-MR results of causal links between gut microbiome and AILD subtypes risk

Gut microbiome	SNPs	OR (95% CI)	*P*-value
**AIH**			
Class_Clostridia	12	0.58 (0.35 to 0.97)	0.039
Family_Victivallaceae	12	1.42 (1.11 to 1.81)	0.005
Genus_Anaerostipes	13	0.35 (0.17 to 0.74)	0.006
Genus_Clostridium innocuum group	9	0.67 (0.49 to 0.93)	0.017
Genus_Holdemania	15	0.66 (0.46 to 0.95)	0.025
Genus_Ruminiclostridium9	9	0.39 (0.20 to 0.76)	0.006
Genus_Ruminococcaceae NK4A214 group	13	1.64 (1.03 to 2.64)	0.039
**PBC**			
Class_Coriobacteriia	3	2.18 (1.30 to 3.66)	0.003
Class_Deltaproteobacteria	4	0.52 (0.36 to 0.74)	< 0.001
Order_Coriobacteriales	3	2.18 (1.30 to 3.66)	0.003
Family_Coriobacteriaceae	3	2.18 (1.30 to 3.66)	0.003
Family_Desulfovibrionaceae	3	0.53 (0.34 to 0.81)	0.003
Genus_Ruminiclostridium5	4	1.47 (1.03 to 2.09)	0.032
**PSC**			
Phylum_Tenericutes	5	1.66 (1.06 to 2.61)	0.027
Class_Mollicutes	5	1.66 (1.06 to 2.61)	0.027
Order_Actinomycetales	3	0.59 (0.36 to 0.98)	0.042
Family_Actinomycetaceae	3	0.60 (0.36 to 0.98)	0.042
Family_Clostridiales vadin BB60 group	10	0.73 (0.54 to 0.98)	0.035
Family_Oxalobacteraceae	5	1.44 (1.08 to 1.91)	0.014
Genus_Actinomyces	4	0.62 (0.42 to 0.90)	0.012
Genus_Alloprevotella	3	0.68 (0.50 to 0.94)	0.018

*OR* odds ratio, *CI* confidence interval

### PBC

The same approaches were utilized to explore the causal effect of gut microbiome on PBC. There were four bacterial traits(*class_Coriobacteriia*: OR 2.18, 95% CI 1.30 to 3.66, *P* = 0.003; *family_Coriobacteriaceae*: OR 2.18, 95% CI 1.30 to 3.66, *P* = 0.003; *order_Coriobacteriales*: OR 2.18, 95% CI 1.30 to 3.66, *P* = 0.003; *genus_Ruminiclostridium5*, OR 1.47, 95% CI 1.03 to 2.09, *P* = 0.031) potentially related to an increased risk of PBC utilizing the IVW method, while two bacterial traits(*class_Deltaproteobacteria*, OR 0.52, 95% CI 0.36 to 0.74, *P* < 0.001; *family_Desulfovibrionaceae*, OR 0.53, 95% CI 0.34 to 0.81, *P* = 0.003) were associated with a lower risk of PBC in the IVW–MR analysis (Table 1). Meanwhile, all the results above were supported by the Weighted Median method (*P* < 0.05, Supplementary Table 2).

### PSC

Similarly, on the causal effect of gut microbiome on PSC, the estimates of the IVW test indicated that certain bacterial traits was associated with either an increased or reduced risk of PSC. Specifically, three bacterial features (*phylum_Tenericutes*: OR 1.66, 95% CI 1.06 to 2.61, *P* = 0.026; *class_Mollicutes*: OR 1.66, 95% CI 1.06 to 2.61, *P* = 0.026; *family_Oxalobacteraceae*, OR 1.44, 95% CI 1.08 to 1.91, *P* = 0.013) were associated with an increased risk of PSC according to IVW analysis. In the contrast, five bacterial features (*order_Actinomycetales*, OR 0.59, 95% CI 0.36 to 0.98, *P* = 0.041; *family_Clostridiales vadin BB60 group*, OR 0.73, 95% CI 0.54 to 0.98, *P* = 0.035; *family_Actinomycetaceae*, OR 0.60, 95% CI 0.36 to 0.98, *P* = 0.042; *genus_Alloprevotella*, OR 0.68, 95% CI 0.50 to 0.94, *P* = 0.018; *genus_Actinomyces* OR 0.62, 95% CI 0.42 to 0.90, *P* = 0.012) were associated with an lower risk of PSC (Table 1). The Weighted Median method provided support for the aggressive role of *family_Oxalobacteraceae* and the protective role of *genus_Alloprevotella*, while other associations did not reach statistical significance (Supplementary Table 2).

In summary, Supplementary Table 2 presents a comprehensive overview of all positive results, providing in-depth details and information. On the other hand, Supplementary Table 3 includes all results, including negative ones, in a tabulated format. These supplementary tables are intended to complement and provide further context to the main findings discussed in the sections above.

### Sensitivity analyses

When the exposure is the *genus*_*Anaerostipes* and the outcome is AIH, the MR result did not pass the heterogeneity test (Q *p*-value = 0.02, Supplementary Table 4). Since there is only heterogeneity and no pleiotropy for this exposure trait, the result of the Weighted Median method are preferred [23]. The value of the Weighted Median method is *P* < 0.001, indicating that causality exists. To increase the credibility of the result, the random effects model of IVW was further performed and the result was *P* = 0.006 < 0.05, which was statistically significant. For other exposure-outcome pairs, no heterogeneity or outliers were found using Cochran’s Q and MR-PRESSO tests (*P* > 0.05, Supplementary Table 4–5). All *P*-values of MR–Egger interpret were > 0.05, indicating no horizontal pleiotropy (Supplementary Table 4). Moreover, Supplementary figures S1-3 show the results of sensitivity analyses in scatter plots. Furthermore, we conducted leave-one-out analyses to evaluate the potential influence of individual SNPs on the observed associations. Supplementary figures S4-6 present the leave-one-out analysis, evaluating the influence of individual SNPs on the associations.

## Discussion

We conducted an MR study using the most comprehensive GWAS data available to overcome a common limitation in epidemiological studies. This could provide important insights into the genetic correlations between the gut microbiome and AILD subtypes. Our results highlighted a causal effect of the abundance of specific bacterial features on the risk of AILD subtypes. To the best of our knowledge, our study is the first to employ the MR framework to investigate the causal relationship between the gut microbiome and AILD. Our discussion primarily focuses on the findings at the genus level, as such an approach is more clinically oriented. Notably, the genera *Anaerostipes*, *Clostridium_innocuum_group*, *Holdemania*, and *Ruminiclostridium9* played a role in protection against AIH, while *Ruminococcaceae_NK4A214_group* increased the risk of AIH. *Ruminiclostridium5* increased the risk of PBC. *Alloprevotella* and *Actinomyces* protected against PSC.

The microbiome's implication in the etiology of autoimmune disorders has garnered substantial attention. The primary mechanism involves immune system deviations mediated through microbial signaling, predominantly via the gut-liver axis [24]. While the implication of microbiota in the pathogenesis of disorders like Type 1 diabetes, rheumatoid arthritis, and coeliac disease has been extensively explored, the literature concerning AILDs remains comparatively limited [25]. However, evidence from animal models underscores a causal connection between dysbiosis of gut microbiota or specific pathobionts and AILDs. For example, gnotobiotic mice administered with microbiota from PSC patients exhibited heightened Th17 cell responses within the liver, rendering them more susceptible to hepatobiliary injuries [26]. This suggests a potential role of gut microbiota in driving PSC pathogenesis. Other research indicates that gut pathobiont translocation, stemming from compromised gut barriers, infiltrates systemic organs in hosts prone to autoimmunity, instigating autoimmune pathogenesis [27].

Several population-based observational studies have been conducted to examine the gut microbiome in patients with AILD [28–30]. Comparing the results to observational studies, we have observed both consistencies and inconsistencies in the association of bacterial traits with AILD. These variations may stem from differences in genetic backgrounds and synergistic activities among populations from different regions. For instance, the *genus_Veillonella* is frequently reported to be enriched in AIH, PBC, and PSC in Asian cohorts [31]. However, our study did not find a causal effect between the *genus_Veillonella* and AILD in the MR analysis. Here, we will specifically examine the potential impact of two traits, namely *genus_Clostridium_innocuum_group* and *genus_Actinomyces* as they exhibited protective role for AIH and PSC respectively. Meanwhile, these two traits were previously reported to be associated with AILD, and the details of the MR analysis pertaining to their effects were provided in Table 2.

**Table 2:** The causal effects of genus_Clostridium_innocuum_group and genus_Actinomyces on AILD subtypes

Gut microbiome	Methods	OR (95% CI)	*P*-value	Q statistic	PQ	Egger intercept	Pintercept
**AIH**							
Genus_Clostridium_innocuum_group	MR Egger	0.63(0.18–2.26)	0.49	5.02	0.66	0.11	0.32
	Weighted median	0.65(0.32–1.33)	0.24				
	Simple mode	0.59(0.22–1.58)	0.31				
	Weighted mode	0.66(0.29–1.48)	0.34				
	Inverse variance weighted	0.58(0.35–0.97)	0.04	8.81	0.64	-0.01	0.9
**PSC**							
Genus_Actinomyces	MR Egger	0.79(0.17–3.59)	0.79	0.99	0.61	-0.03	0.77
	Weighted median	0.66(0.42–1.03)	0.07				
	Simple mode	0.70(0.37–1.31)	0.34				
	Weighted mode	0.71(0.39–1.30)	0.35				
	Inverse variance weighted	0.62(0.42–0.90)	0.01	1.1	0.78	-0.03	0.77

*OR* odds ratio, *CI* confidence interval, *PQ* *p*-value of Q test. Cochran’s Q tests heterogeneity; MR-Egger detects directional pleiotropy

So far, there are relatively few studies about the relationship between gut microbiome and AIH. In the current study, the *genus_Clostridium_innocuum_group* could mitigate the risk of AIH. It belongs to the *order_Clostridiales* and the latter has been adapted as a biomarker to distinguish AIH from controls in a microbial diagnostic model [28]. It is also reported that the *genus_Clostridium* was more abundant in all subgroups of PBC and PSC [32, 33], however, in our study, no causal effect has been revealed between the *genus_Clostridium* and these subtypes, though a group of *family_Clostridiales* exhibited protective role in our MR analysis for PSC. Moreover, the *genus_Clostridium* has been found to modulate the induction of T regulatory cells through the provision of bacterial antigens and short-chain fatty acids [34]. These factors influence the activity of T regulatory cells and contribute to the reduction of pro-inflammatory cytokine levels [35].

Evidence indicates an elevated relative abundance of *genus_Actinomyces* in both saliva and fecal samples of PSC patients [36, 37]. Our findings suggest that the genus_*Actinomyces* may play a protective role in PSC patients, further supporting these findings. In contrast, the genus_Actinomyces was observed to be lower in AIH patients than in healthy controls [38]. Therefore, the role of the *genus_Actinomyces* in different subtypes of AILD may differ, and further investigation is required.

Notably, our MR analysis revealed contrasting findings regarding the *genus_Ruminiclostridium* in relation to AIH and PBC. The presence of *genus_Ruminiclostridium5* was associated with an increased risk of PBC, while the *genus_Ruminiclostridium9*, another unidentified group within the *genus_Ruminiclostridium*, exhibited a protective effect with greater statistical significance in AIH. The *genus_Ruminiclostridium* is known to be involved in glucose and bile acid metabolism [39]. Considering that a subset of patients (2–19%) may exhibit overlapping features of both PBC and AIH, known as PBC-AIH overlap syndrome [40], caution should be exercised in interpreting these results. Further investigation is warranted to elucidate the role of this microbial trait in the pathogenesis of these subtypes.

The investigation of alterations in the gut microbiome holds significant clinical implications for AILD. First, changes in the gut microbiome can serve as a biomarker for disease screening, diagnosis, and prognosis throughout the course of AILD. The human microbiome has been successfully utilized to develop diagnostic biomarkers for various diseases, including hepatocellular carcinoma [41]. In the context of AILD, diagnostic models based on the microbiome have been established for AIH [28] and PSC [42], but there is currently no reported model specifically for PBC. In the future, there is a need for more longitudinal data on the gut microbiome to support the development of screening, diagnosis, and prognosis models for AILD.

Second, current clinical approaches for treating AILD are limited. Standard therapy for AIH involves a combination of prednisone and azathioprine [43], while ursodeoxycholic acid (UDCA) is commonly used for PBC and PSC. However, the efficacy of UDCA in improving survival in PSC is uncertain, and higher doses are associated with increased adverse events [44]. Therefore, understanding the causal relationship between the gut microbiome and the development and progression of AILD is of great significance in identifying new therapeutic targets and drugs. Probiotics have shown potential as a promising adjunctive therapeutic option in the management of AILD [45]. In the routine management of patients, incorporating food products containing beneficial bacterial components into their daily diet is a relatively easy to implement, cost-effective, and efficient approach. Probiotics were found to increase the population of T regulatory cells in AIH mouse model, indicating their immunomodulatory role in alleviating autoimmune hepatitis [46]. The therapeutic potential of *Lactobacillus* in combination with prednisone for the treatment of AIH has been suggested from a clinical trait [47]. However, a randomized, placebo-controlled study of probiotics in patients with PSC did not show any benefits in relieving PSC symptoms, indicating that probiotics alone may not be effective in treating PSC [48].

Limitations of our research include the potential influence of various factors on the abundance of the gut microbiome, such as diet, sex, medication, and sampling time. To obtain more comprehensive results, we refrained from applying a strict false discovery rate correction to re-evaluate positive outcomes. Future research demands more rigorous experimental and clinical validation of our findings. Furthermore, it underscores the necessity for comprehensive GWAS tailored to Asian populations to delve into host genetic variants associated with the gut microbiome [49]. Additionally, the overlapping symptoms among different subtypes of AILD can complicate the diagnosis process, making it more relevant to compare results across subtypes rather than relying solely on subtype-specific conclusions to draw definitive causal inferences regarding the relationship between the gut microbiome and AILD.

## Conclusion

Our study provides evidence supporting the causal effect of specific bacterial features on the risk of AILD subtypes. Specifically, we found that the *genus_Clostridium_innocuum_group* displayed a significant protective effect against AIH, while the *genus_Actinomyces* showed a significant protective effect against PSC. Further longitudinal studies and clinical trials are needed to validate these findings and explore the potential of targeted probiotics for the management of AILD.

### Supplementary Information

**Additional file 1:** **Supplementary Figure S1-3.** Results of sensitivity analyses displayed in scatter plots for AIH (S1), PBC (S2) and PSC (S3). **Supplementary Figure S4-6.** Results of leave-one-out analyses, evaluating the influence of individual SNPs on the associations for AIH (S4), PBC (S5) and PSC (S6). **Supplementary Table S1.** Instrumental Variables for each baterial triats. **Supplementary Table S2.** Positive results of MR analyses. **Supplementary Table S3.** All MR results for 194 traits. **Supplementary Table S4.** Results of sensitivity analyses for IVW positive MR analyses. **Supplementary Table S5.** Results of MR-PRESSO.

## Data Availability

All data generated or analyzed during this study are included in this published article and its supplementary files. The accession number for GWAS data of AIH is GCST90018785, available at http://ftp.ebi.ac.uk/pub/databases/gwas/summary_statistics/GCST90018001-GCST90019000/GCST90018785/GCST90018785_buildGRCh37.tsv.gz). The accession number for PBC is GCST90061440, available at http://ftp.ebi.ac.uk/pub/databases/gwas/summary_statistics/GCST90061001-GCST90062000/GCST90061440/GCST90061440_buildGRCh37.tsv, and accession number for PSC is GCST004030, available at http://ftp.ebi.ac.uk/pub/databases/gwas/summary_statistics/GCST004001-GCST005000/GCST004030/harmonised/27992413-GCST004030-EFO_0004268-Build37.f.tsv.gz.

## Abbreviations

AIH: Autoimmune hepatitis
AILD: Autoimmune liver disease
CI: Confidence interval
GWAS: Genome-Wide Association Study
IVW: Inverse variance-weighted
IV: Instrumental variable
MR: Mendelian randomization
MR-PRESSO: MR-Pleiotropy Residual Sum and Outlier
OR: Odds ratio
PBC: Primary biliary cholangitis
PSC: Primary biliary cholangitis
SNP: Single nucleotide polymorphism
UDCA: Ursodeoxycholic acid
