# Supplementary material for: Causal effects of gut microbiome on autoimmune liver disease: a two-sample Mendelian randomization study
Source: BMC Med Genomics. 2023 Oct 3;16:232. doi: 10.1186/s12920-023-01670-0 (PMC10548566; doi:10.1186/s12920-023-01670-0)
Supplement: Supplementary file 1 — Additional file 1: Supplementary Figure S1-3. Results of sensitivity analyses displayed in scatter plots for AIH (S1), PBC (S2) and PSC (S3). Supplementary Figure S4-6. Results of leave-one-out analyses, evaluating the influence of individual SNPs on the associations for AIH (S4), PBC (S5) and PSC (S6). Supplementary Table S1. Instrumental Variables for each baterial triats. Supplementary Table S2. Positive results of MR analyses. Supplementary Table S3. All MR results for 194 traits. Supplementary Table S4. Results of sensitivity analyses for IVW positive MR analyses. Supplementary Table S5. Results of MR-PRESSO. [file 12920_2023_1670_MOESM1_ESM.zip › Supplemental Materials/figs/S3 scatterplot_psc.pdf]

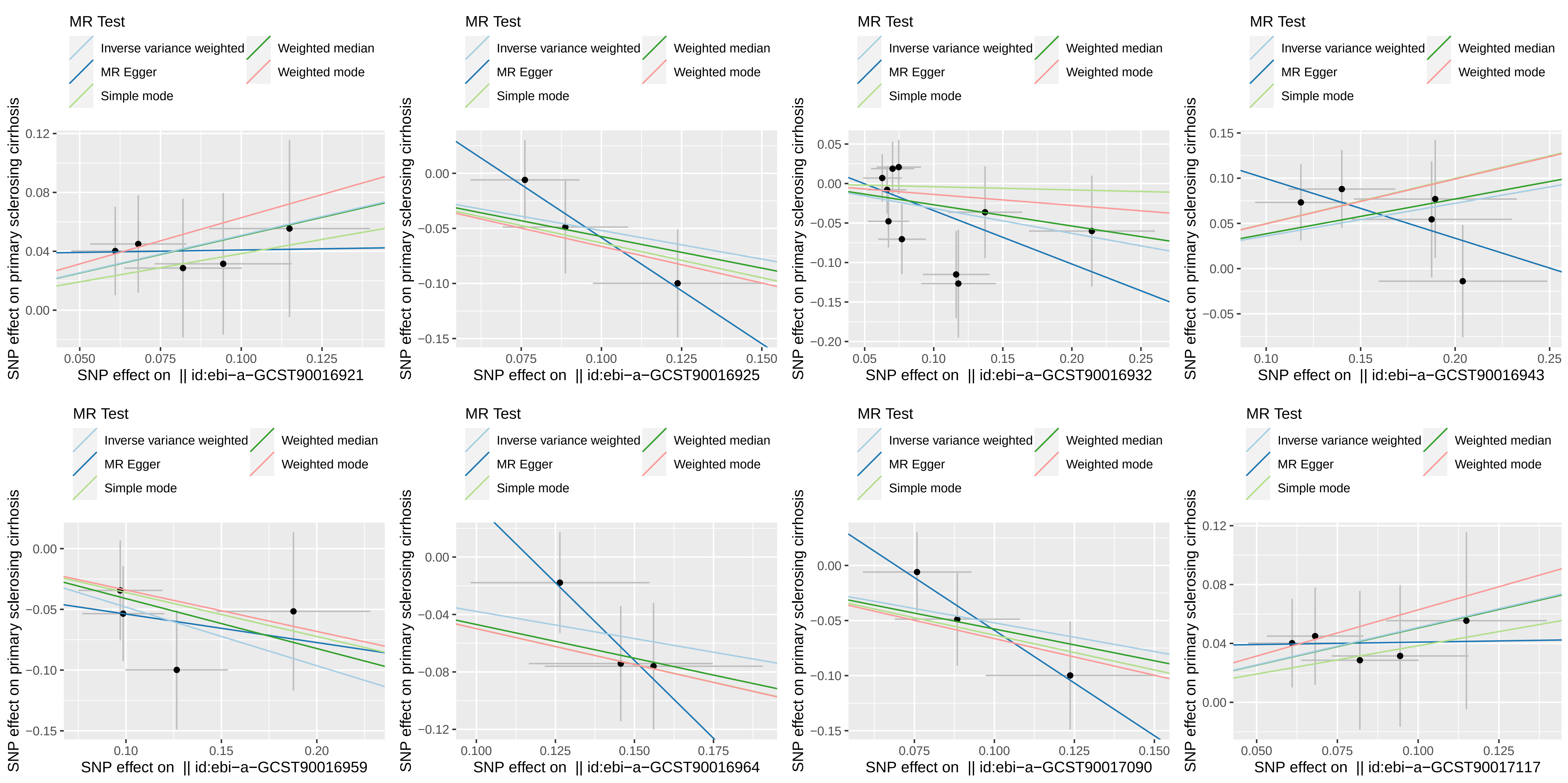
MR Test
Inverse variance weighted
MR Egger
Simple mode
Weighted median
Weighted mode
SNP effect on primary sclerosing cirrhosis
0.12
0.08
0.04
0.00
0.050
0.075
0.100
0.125
SNP effect on || id:ebi−a−GCST90016921
MR Test
Inverse variance weighted
MR Egger
Simple mode
Weighted median
Weighted mode
SNP effect on primary sclerosing cirrhosis
0.00
−0.05
−0.10
−0.15
0.075
0.100
0.125
0.150
SNP effect on || id:ebi−a−GCST90016925
MR Test
Inverse variance weighted
MR Egger
Simple mode
Weighted median
Weighted mode
SNP effect on primary sclerosing cirrhosis
0.05
0.00
−0.05
−0.10
−0.15
−0.20
0.05
0.10
0.15
0.20
0.25
SNP effect on || id:ebi−a−GCST90016932
MR Test
Inverse variance weighted
MR Egger
Simple mode
Weighted median
Weighted mode
SNP effect on primary sclerosing cirrhosis
0.15
0.10
0.05
0.00
−0.05
0.10
0.15
0.20
0.25
SNP effect on || id:ebi−a−GCST90016943
MR Test
Inverse variance weighted
MR Egger
Simple mode
Weighted median
Weighted mode
SNP effect on primary sclerosing cirrhosis
0.00
−0.05
−0.10
−0.15
0.10
0.15
0.20
SNP effect on || id:ebi−a−GCST90016959
MR Test
Inverse variance weighted
MR Egger
Simple mode
Weighted median
Weighted mode
SNP effect on primary sclerosing cirrhosis
0.00
−0.04
−0.08
−0.12
0.100
0.125
0.150
0.175
SNP effect on || id:ebi−a−GCST90016964
MR Test
Inverse variance weighted
MR Egger
Simple mode
Weighted median
Weighted mode
SNP effect on primary sclerosing cirrhosis
0.00
−0.05
−0.10
−0.15
0.075
0.100
0.125
0.150
SNP effect on || id:ebi−a−GCST90017090
MR Test
Inverse variance weighted
MR Egger
Simple mode
Weighted median
Weighted mode
SNP effect on primary sclerosing cirrhosis
0.12
0.08
0.04
0.00
0.050
0.075
0.100
0.125
SNP effect on || id:ebi−a−GCST90017117
