# Supplementary figures and images for: Causal effects of gut microbiome on autoimmune liver disease: a two-sample Mendelian randomization study
Source: BMC Med Genomics. 2023 Oct 3;16:232. doi: 10.1186/s12920-023-01670-0 (PMC10548566; doi:10.1186/s12920-023-01670-0)

SNP effect on autoimmune hepatitis

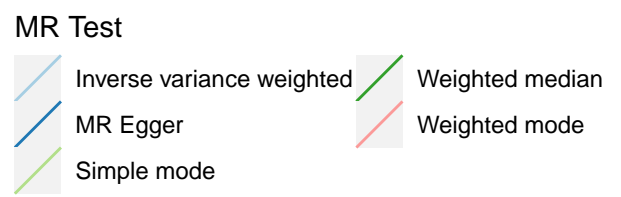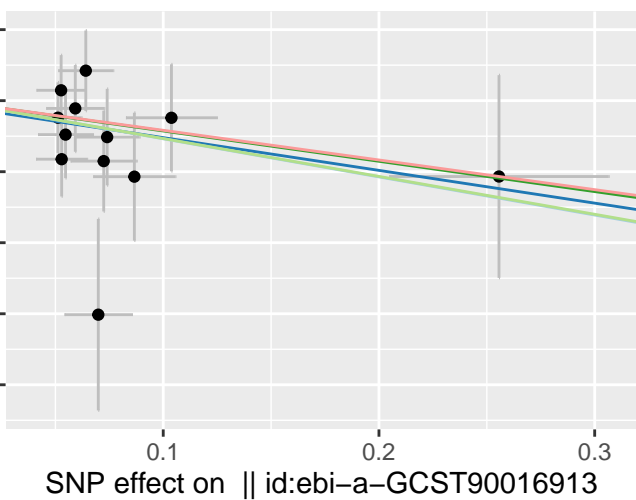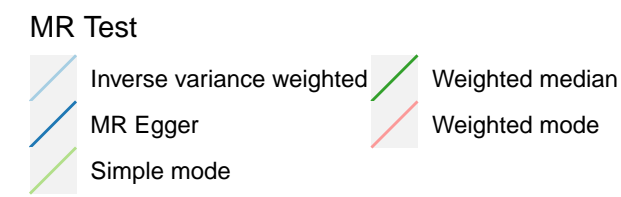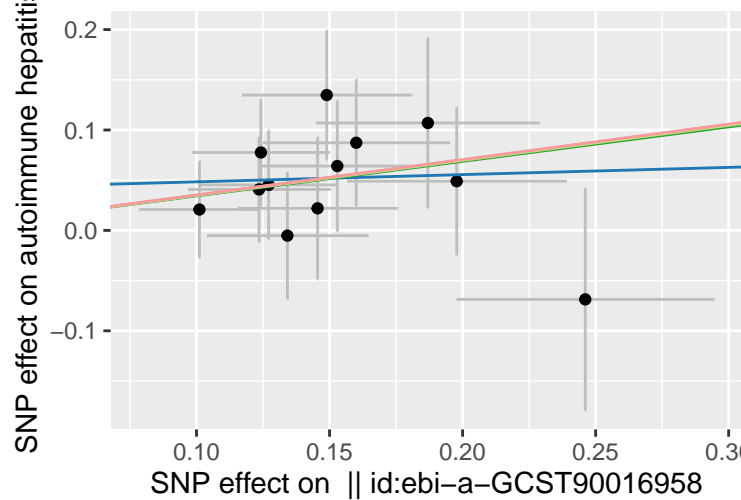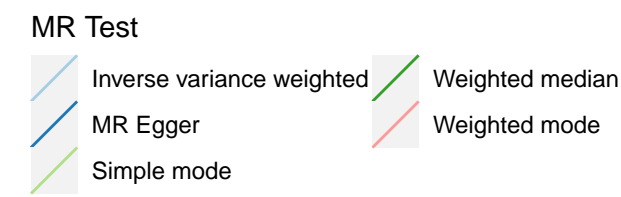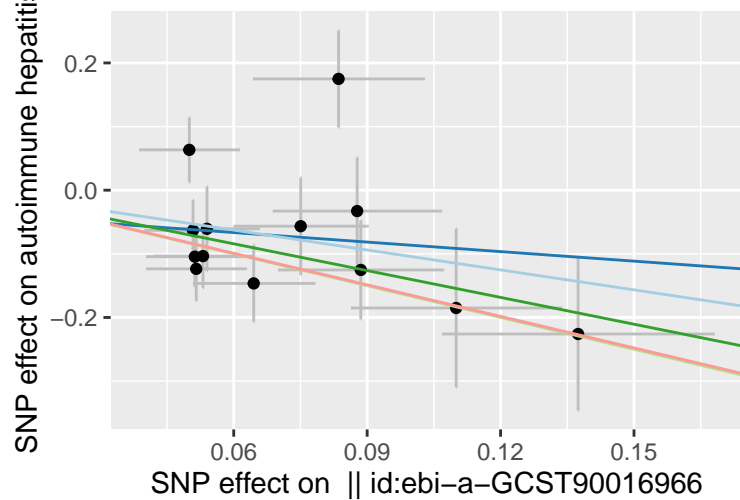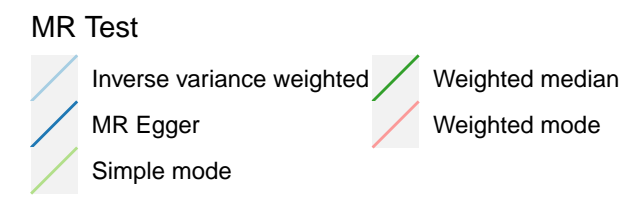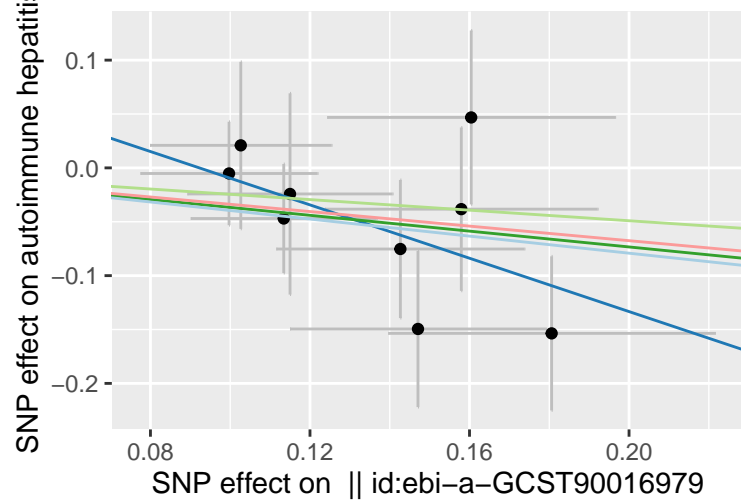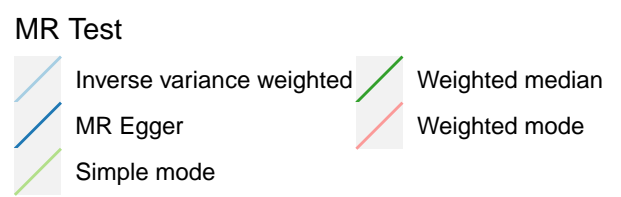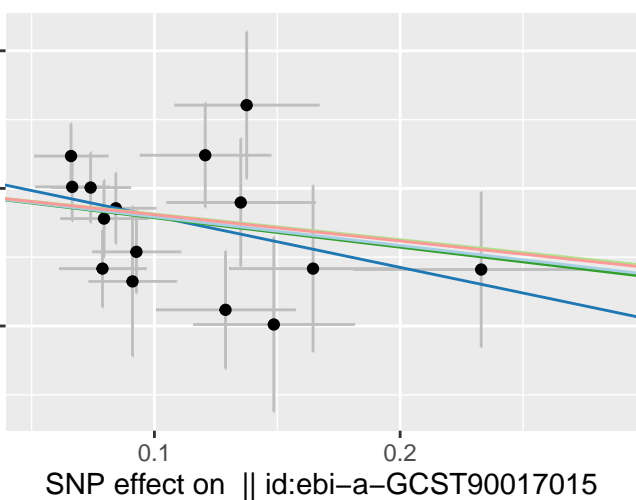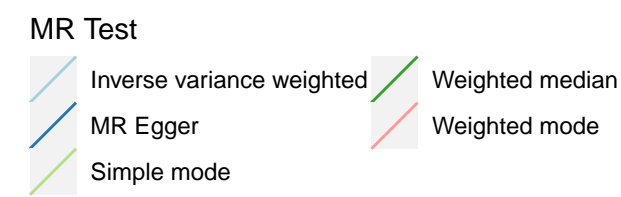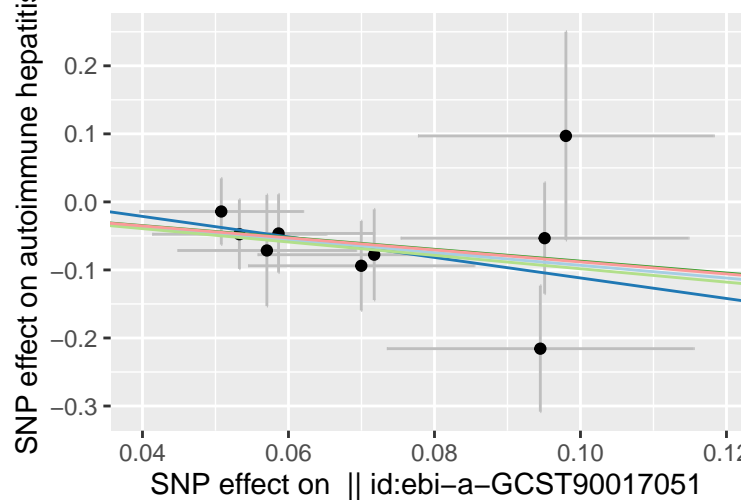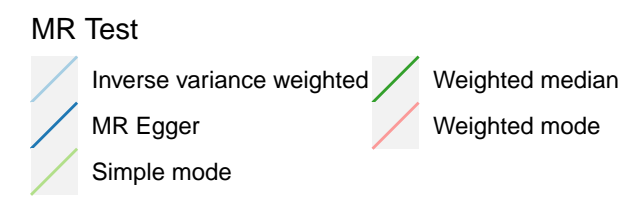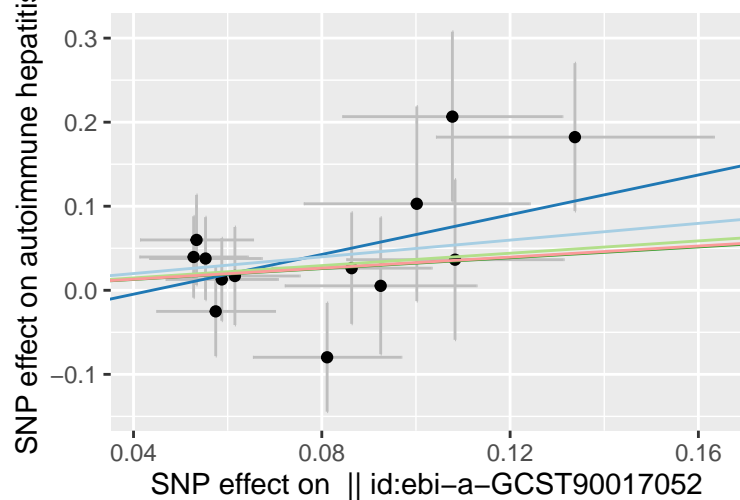

Supplement: Supplementary file 1 — Additional file 1: Supplementary Figure S1-3. Results of sensitivity analyses displayed in scatter plots for AIH (S1), PBC (S2) and PSC (S3). Supplementary Figure S4-6. Results of leave-one-out analyses, evaluating the influence of individual SNPs on the associations for AIH (S4), PBC (S5) and PSC (S6). Supplementary Table S1. Instrumental Variables for each baterial triats. Supplementary Table S2. Positive results of MR analyses. Supplementary Table S3. All MR results for 194 traits. Supplementary Table S4. Results of sensitivity analyses for IVW positive MR analyses. Supplementary Table S5. Results of MR-PRESSO. [file 12920_2023_1670_MOESM1_ESM.zip › Supplemental Materials/figs/S1 scatterplot_aih.pdf]

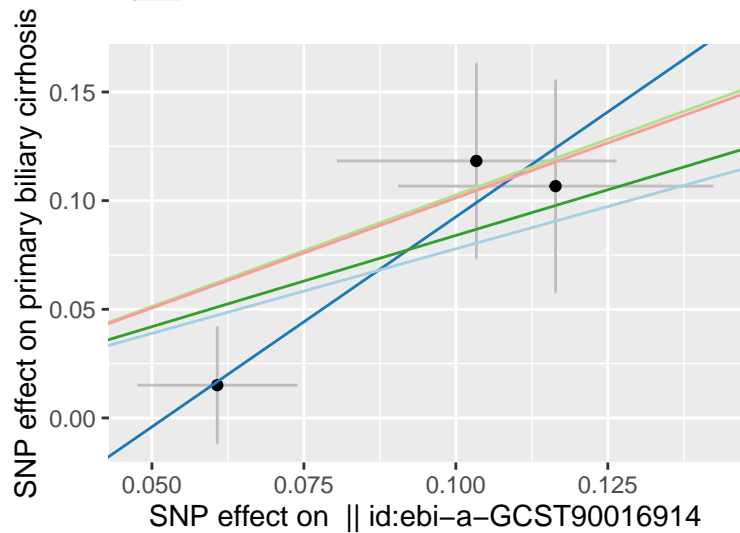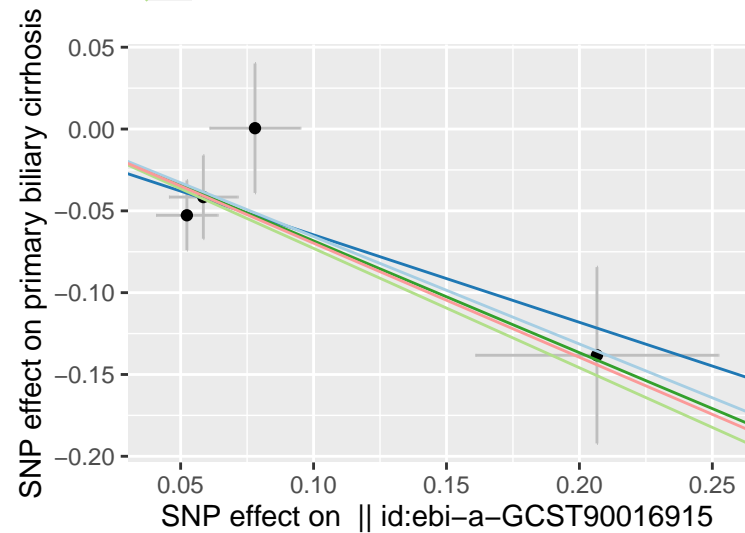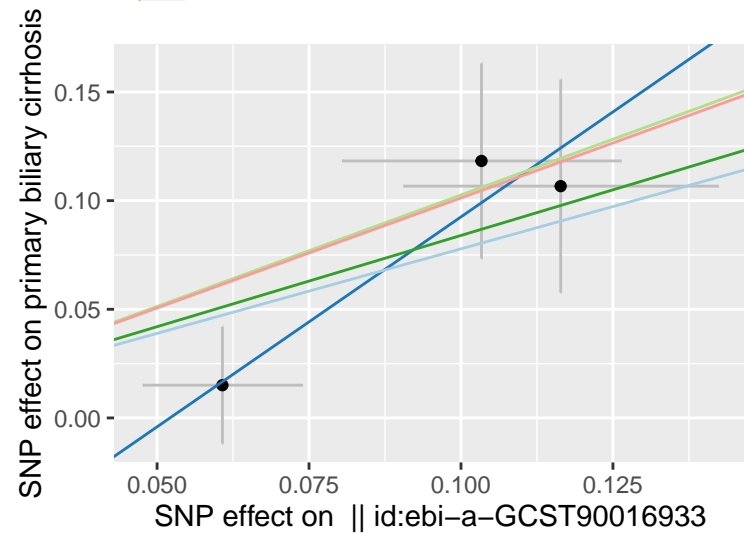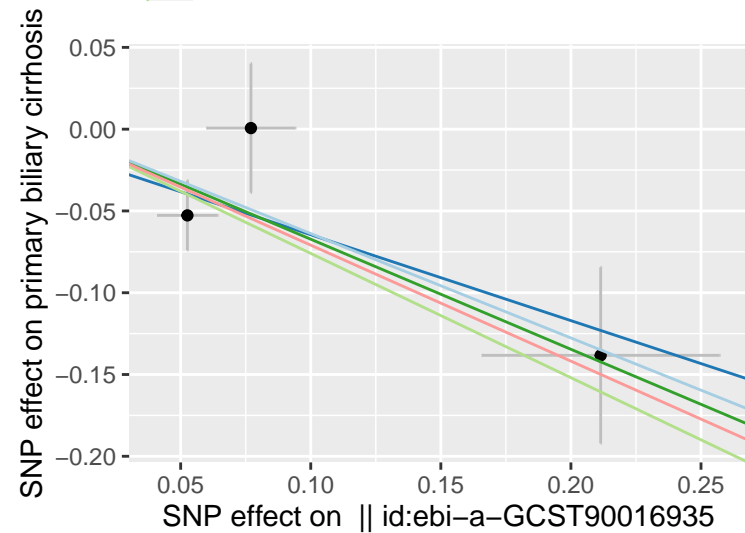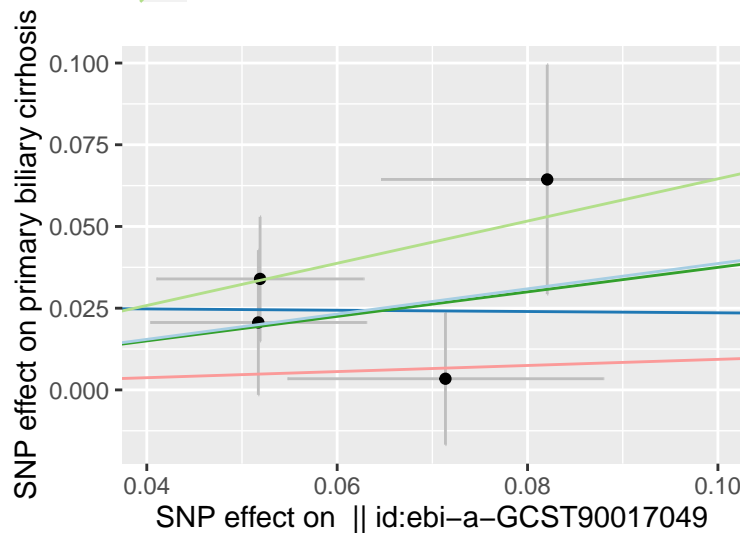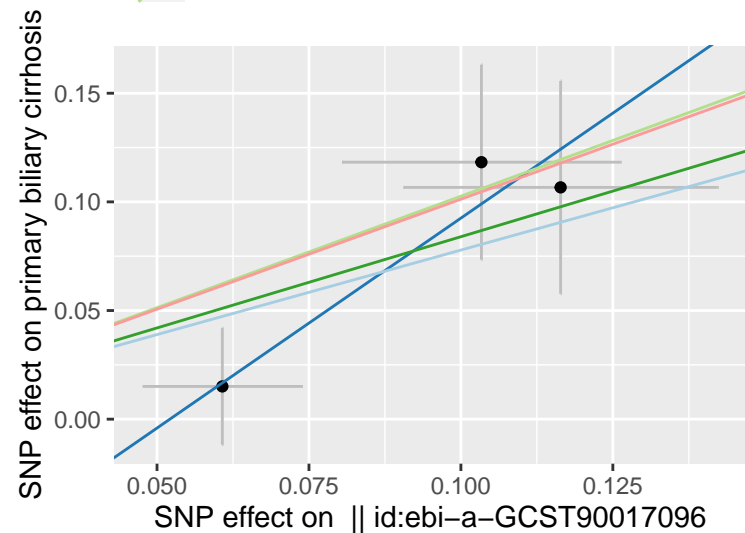

Supplement: Supplementary file 1 — Additional file 1: Supplementary Figure S1-3. Results of sensitivity analyses displayed in scatter plots for AIH (S1), PBC (S2) and PSC (S3). Supplementary Figure S4-6. Results of leave-one-out analyses, evaluating the influence of individual SNPs on the associations for AIH (S4), PBC (S5) and PSC (S6). Supplementary Table S1. Instrumental Variables for each baterial triats. Supplementary Table S2. Positive results of MR analyses. Supplementary Table S3. All MR results for 194 traits. Supplementary Table S4. Results of sensitivity analyses for IVW positive MR analyses. Supplementary Table S5. Results of MR-PRESSO. [file 12920_2023_1670_MOESM1_ESM.zip › Supplemental Materials/figs/S2 scatterplot_pbc.pdf]

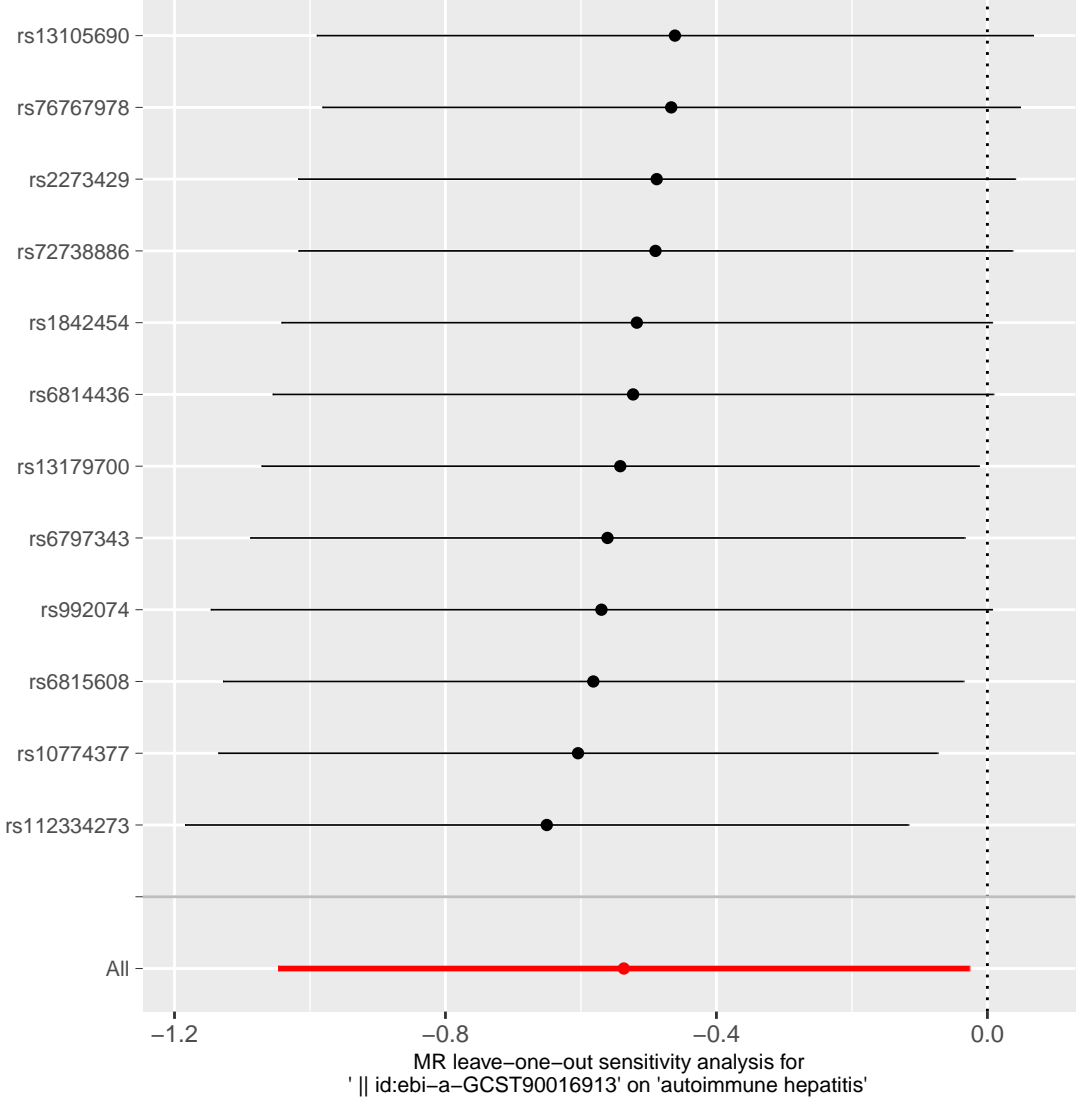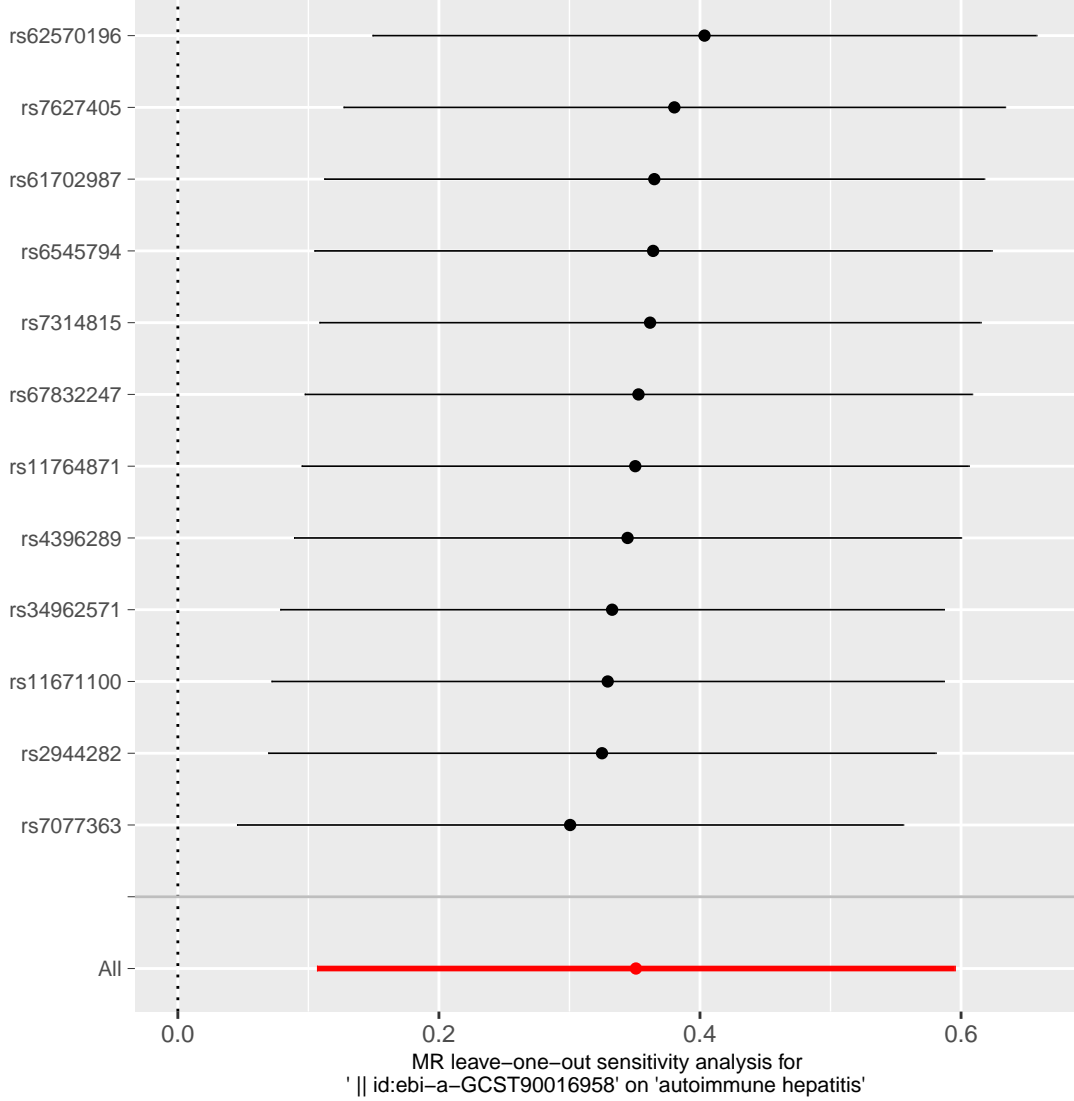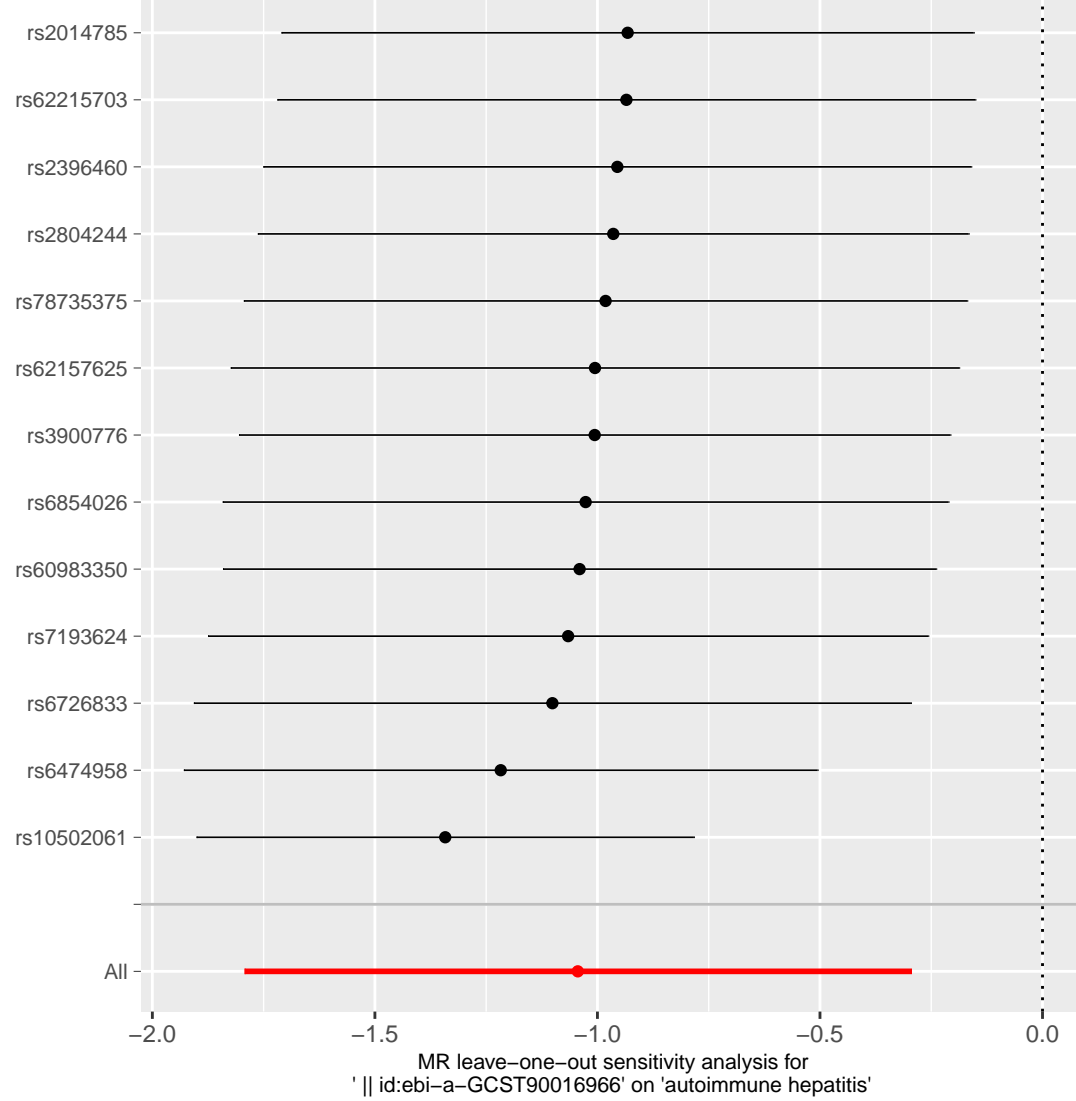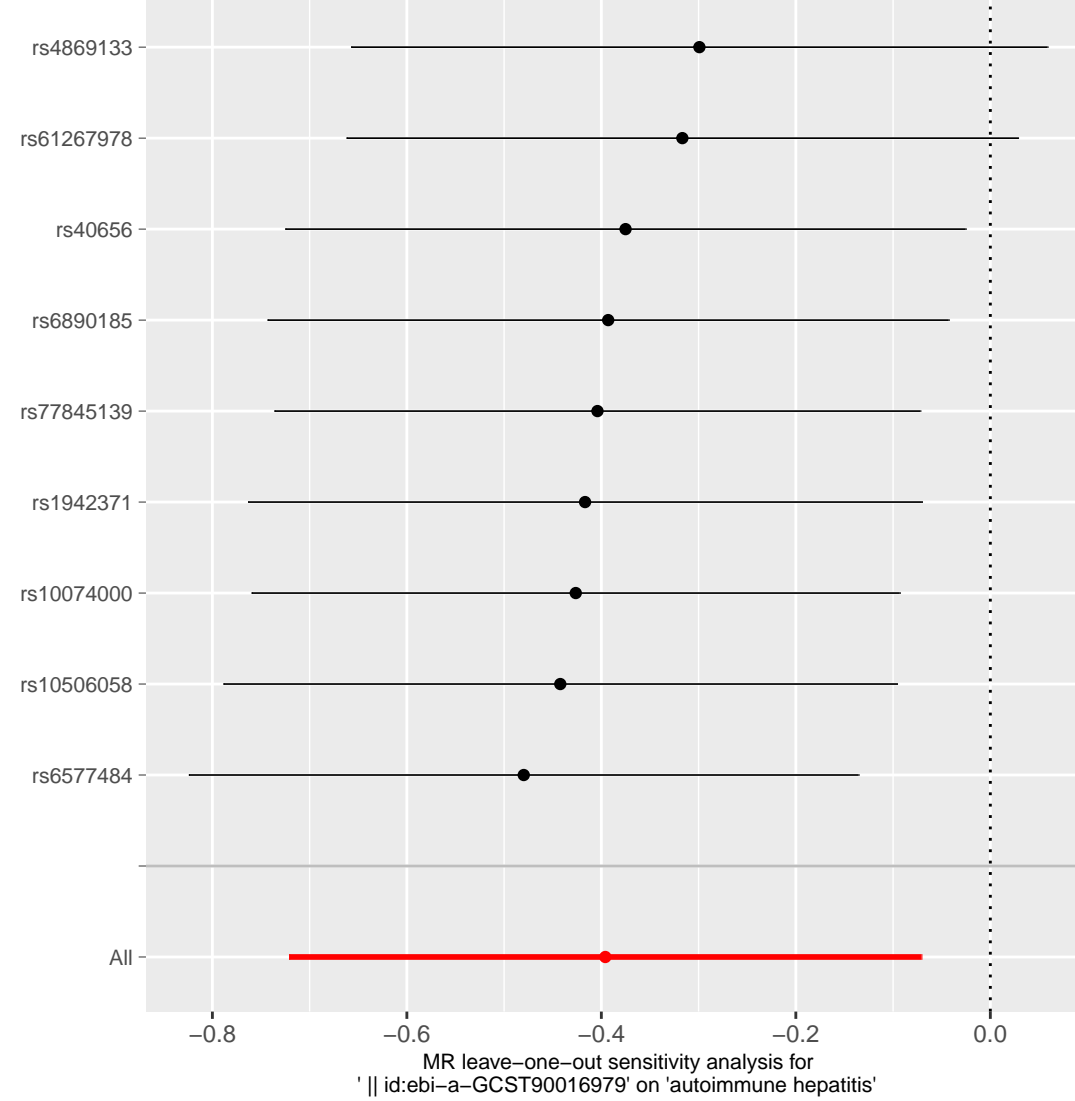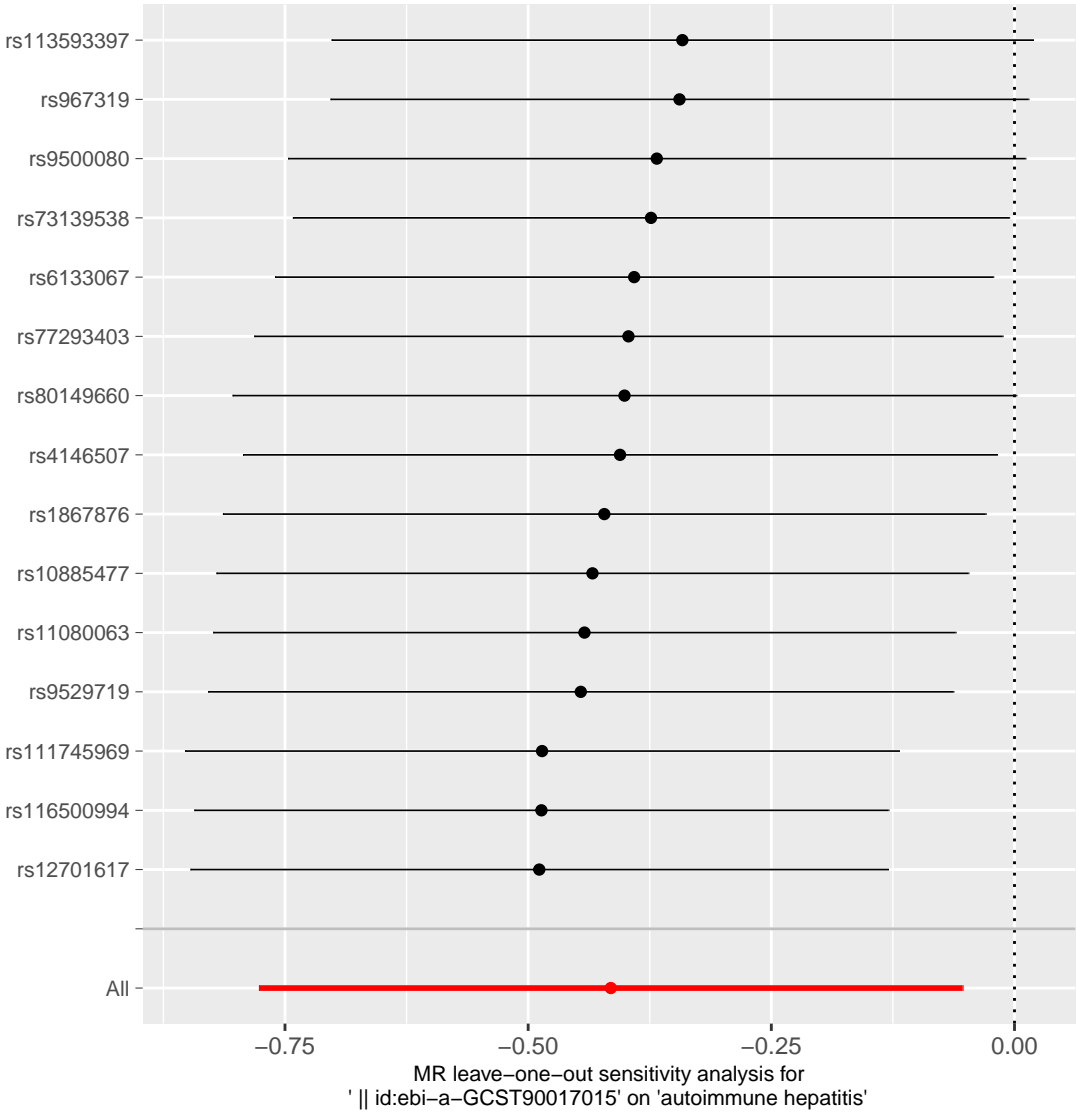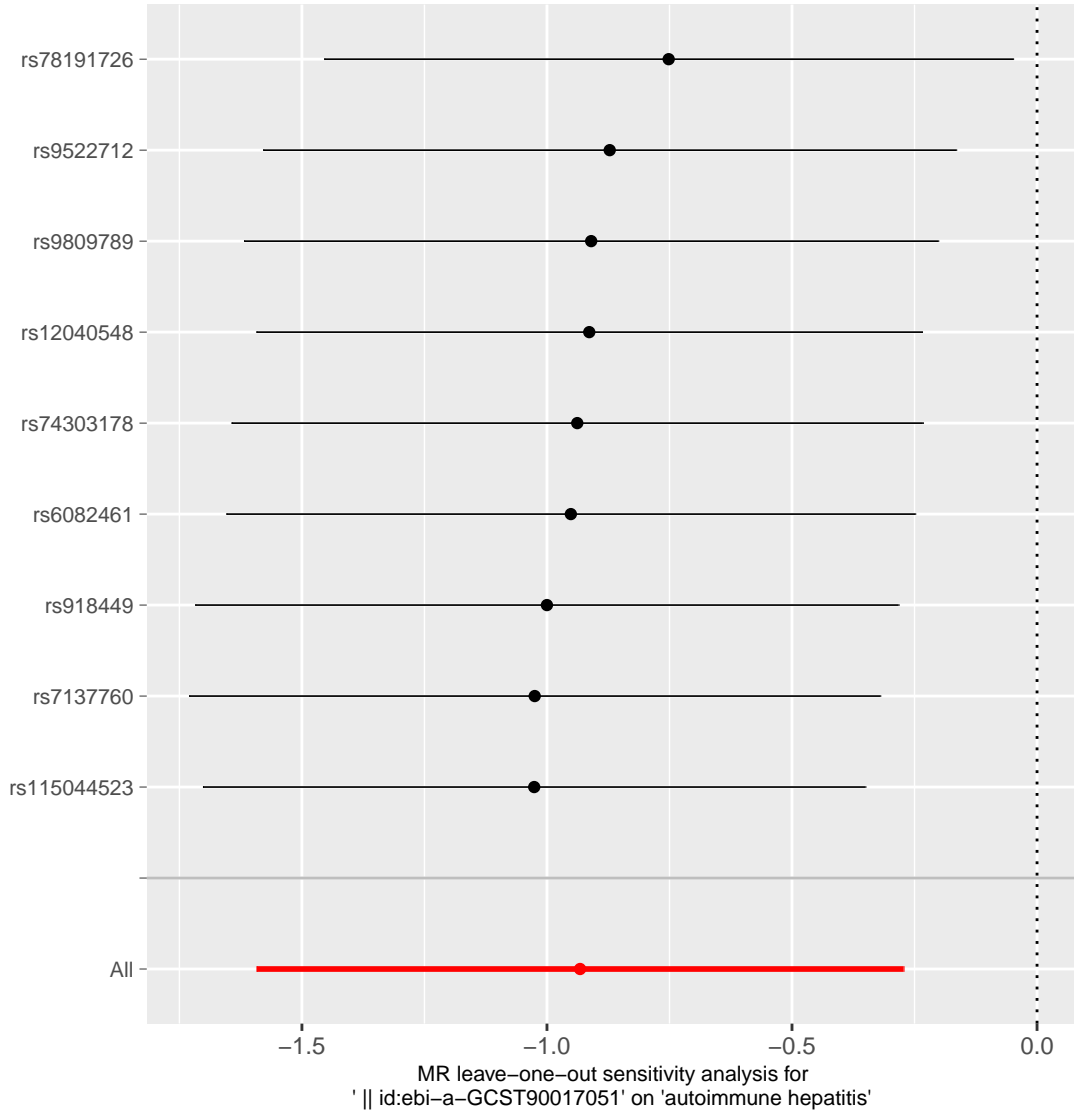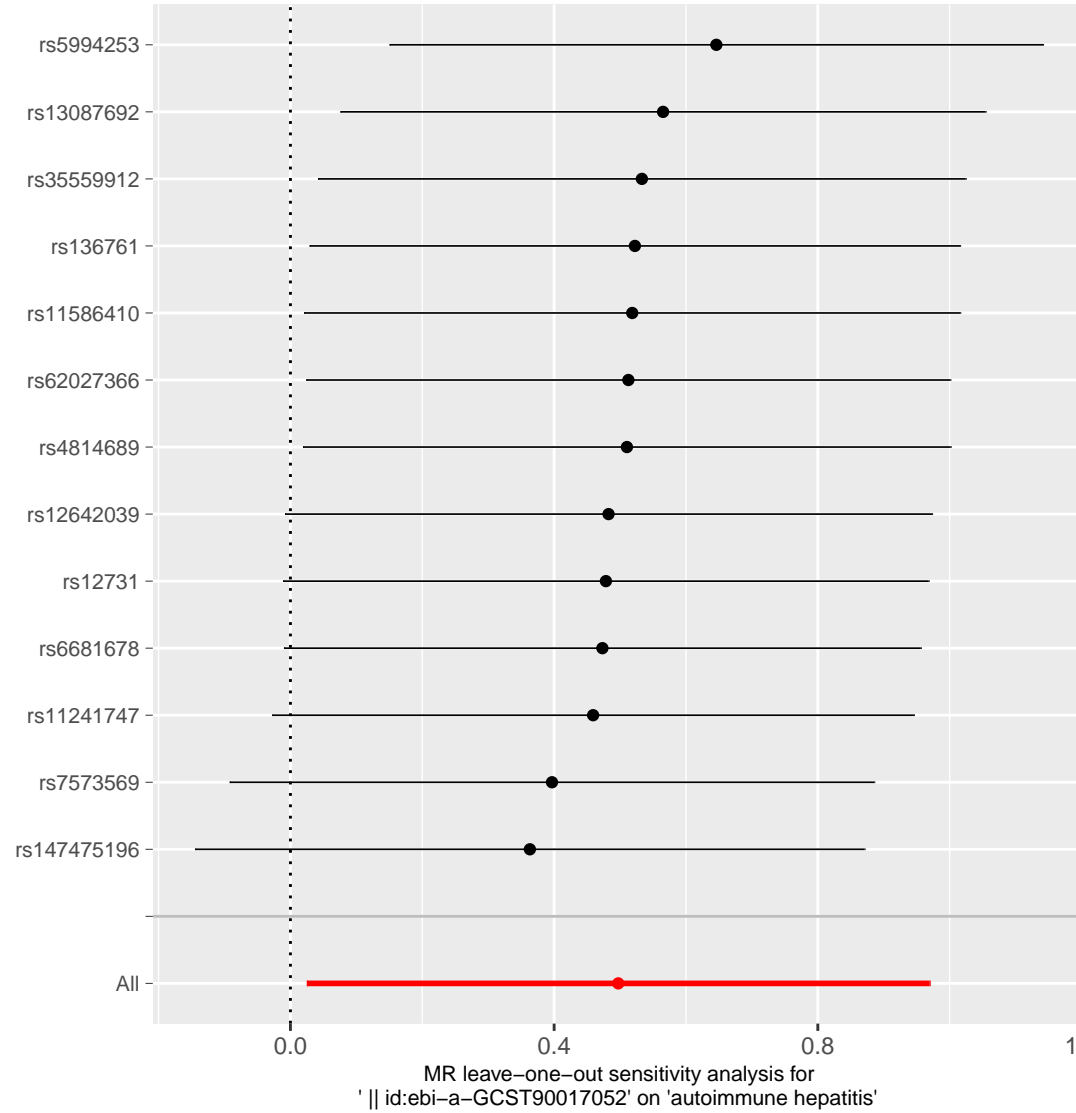

Supplement: Supplementary file 1 — Additional file 1: Supplementary Figure S1-3. Results of sensitivity analyses displayed in scatter plots for AIH (S1), PBC (S2) and PSC (S3). Supplementary Figure S4-6. Results of leave-one-out analyses, evaluating the influence of individual SNPs on the associations for AIH (S4), PBC (S5) and PSC (S6). Supplementary Table S1. Instrumental Variables for each baterial triats. Supplementary Table S2. Positive results of MR analyses. Supplementary Table S3. All MR results for 194 traits. Supplementary Table S4. Results of sensitivity analyses for IVW positive MR analyses. Supplementary Table S5. Results of MR-PRESSO. [file 12920_2023_1670_MOESM1_ESM.zip › Supplemental Materials/figs/S4 leaveoutplot_aih.pdf]

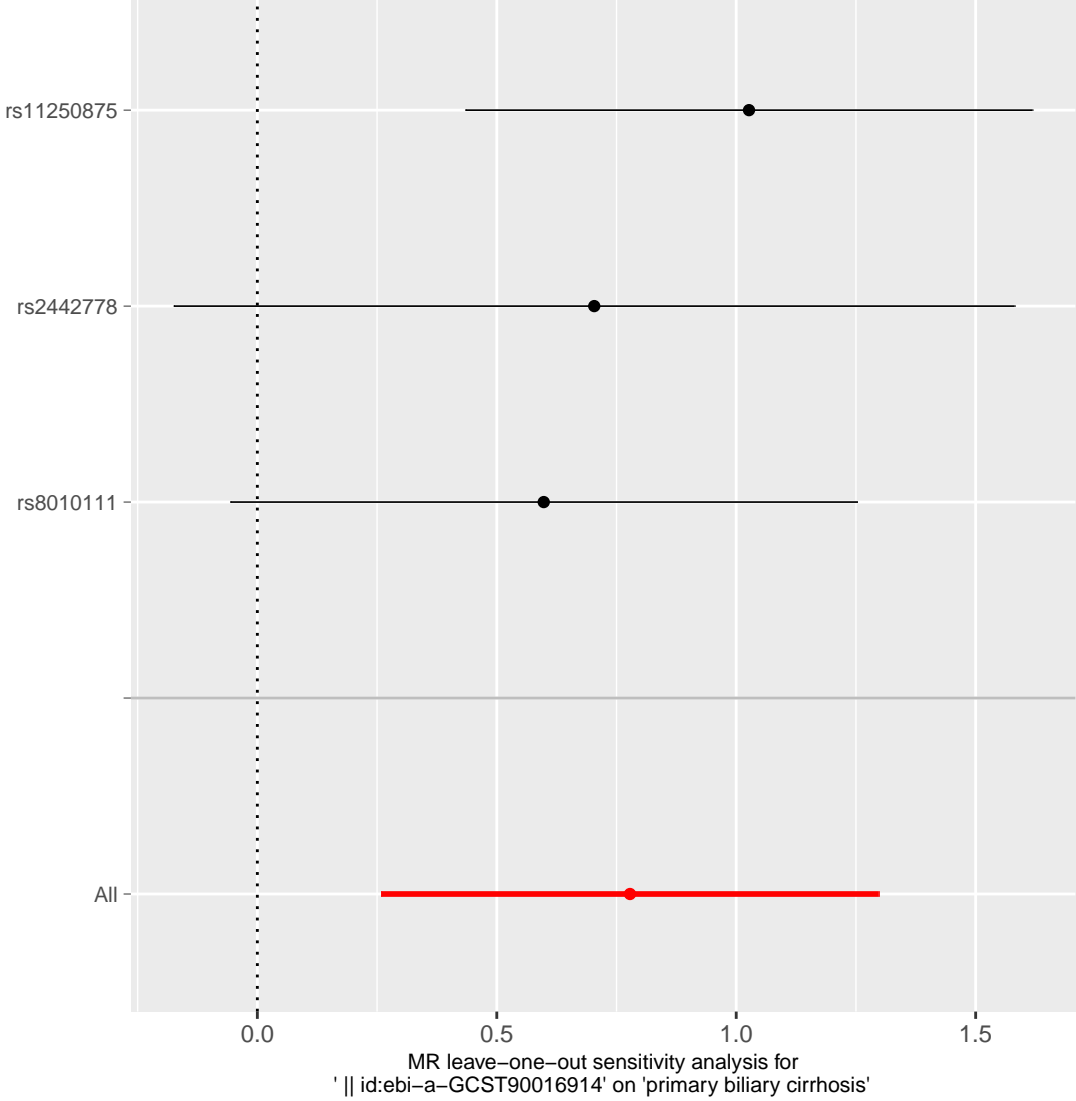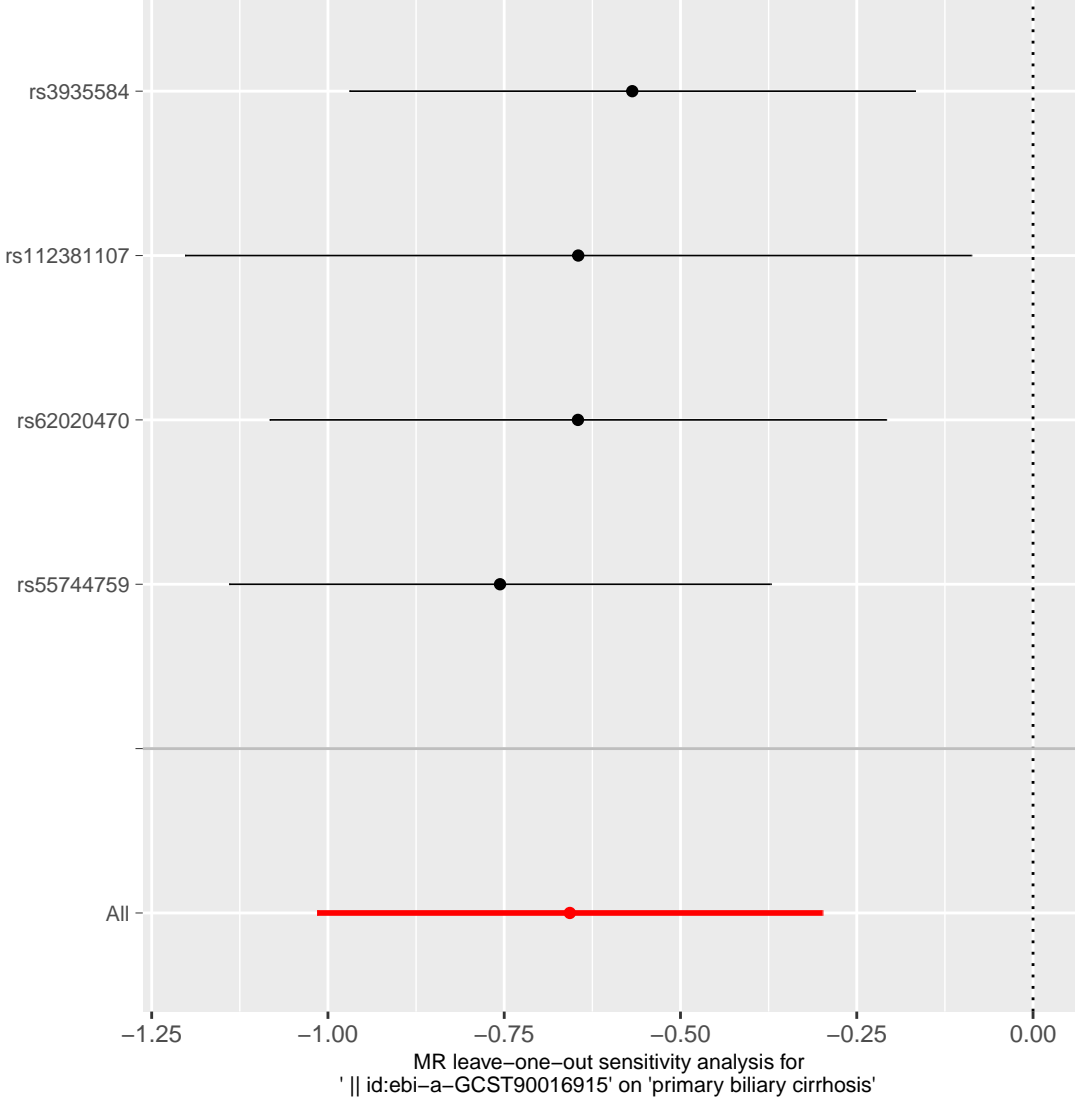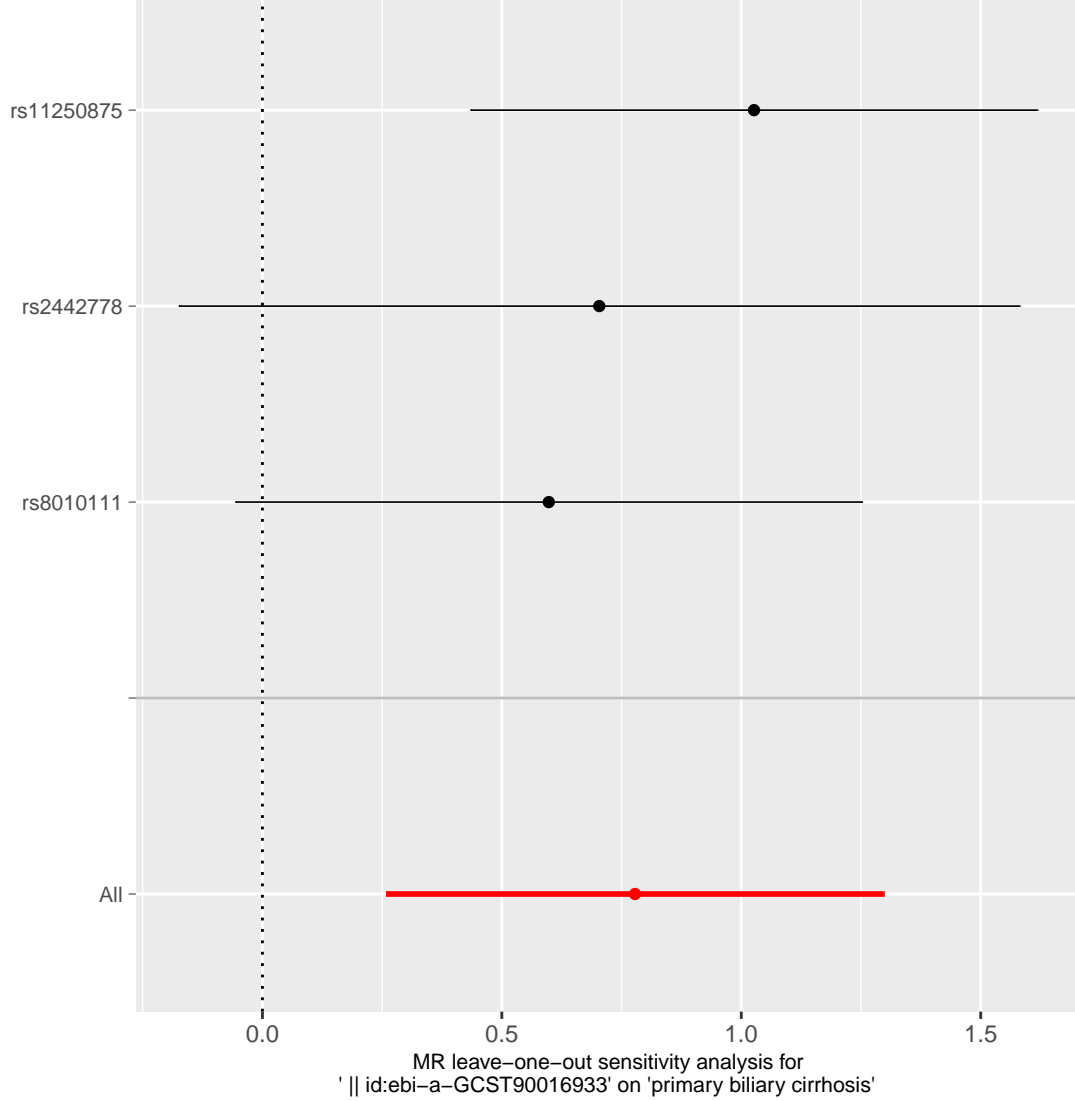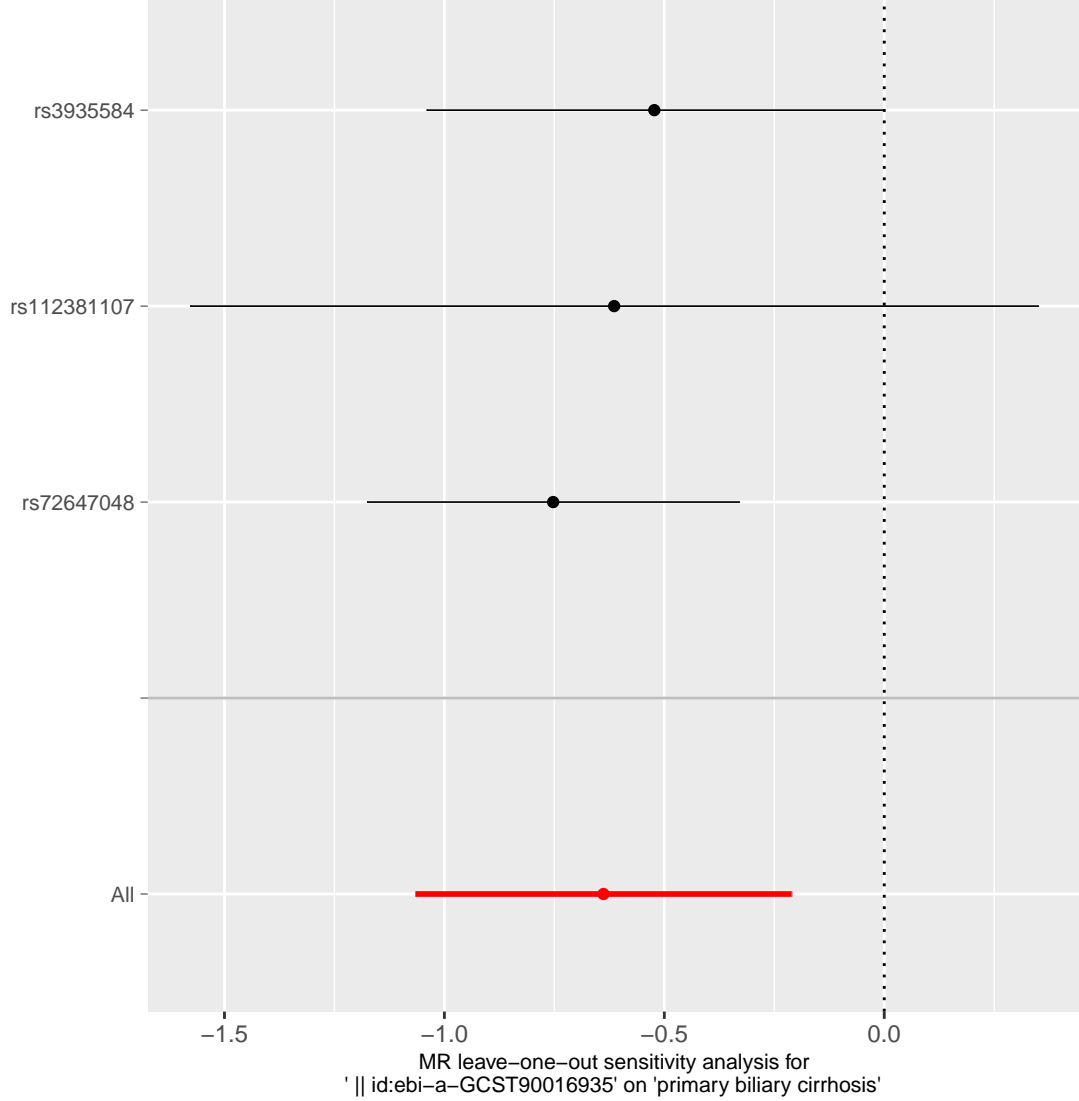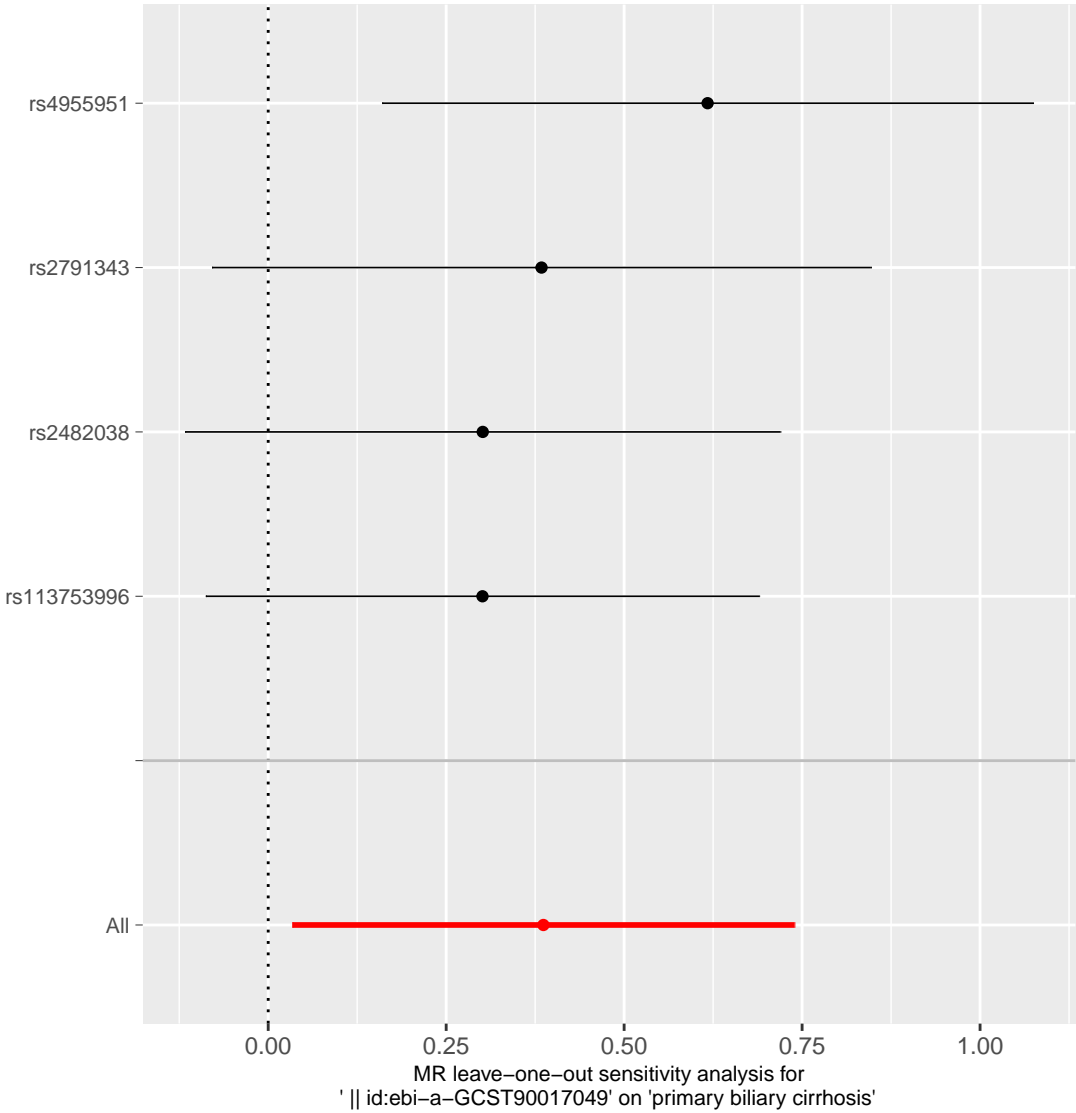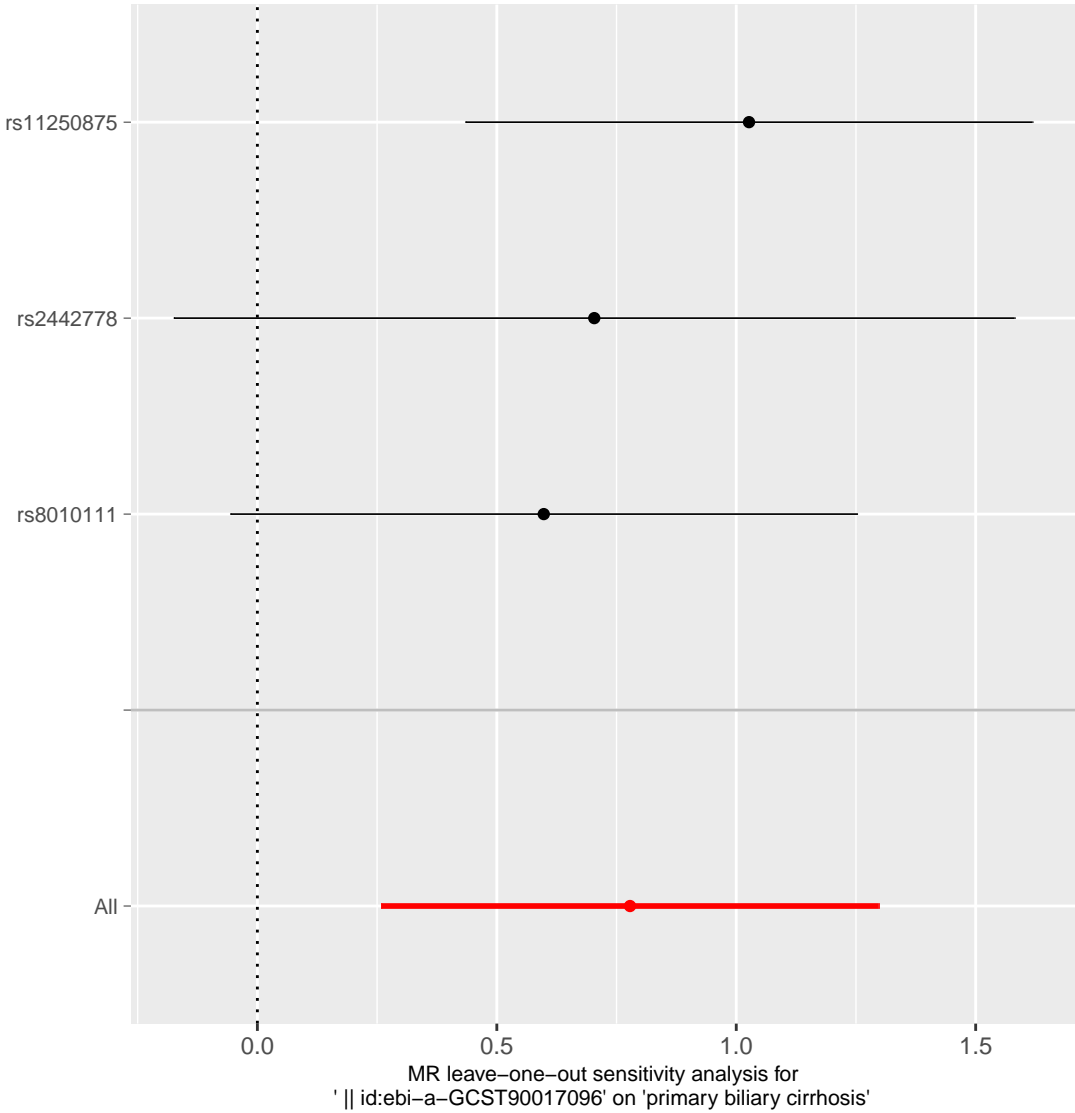

Supplement: Supplementary file 1 — Additional file 1: Supplementary Figure S1-3. Results of sensitivity analyses displayed in scatter plots for AIH (S1), PBC (S2) and PSC (S3). Supplementary Figure S4-6. Results of leave-one-out analyses, evaluating the influence of individual SNPs on the associations for AIH (S4), PBC (S5) and PSC (S6). Supplementary Table S1. Instrumental Variables for each baterial triats. Supplementary Table S2. Positive results of MR analyses. Supplementary Table S3. All MR results for 194 traits. Supplementary Table S4. Results of sensitivity analyses for IVW positive MR analyses. Supplementary Table S5. Results of MR-PRESSO. [file 12920_2023_1670_MOESM1_ESM.zip › Supplemental Materials/figs/S5 leaveoutplot_pbc.pdf]

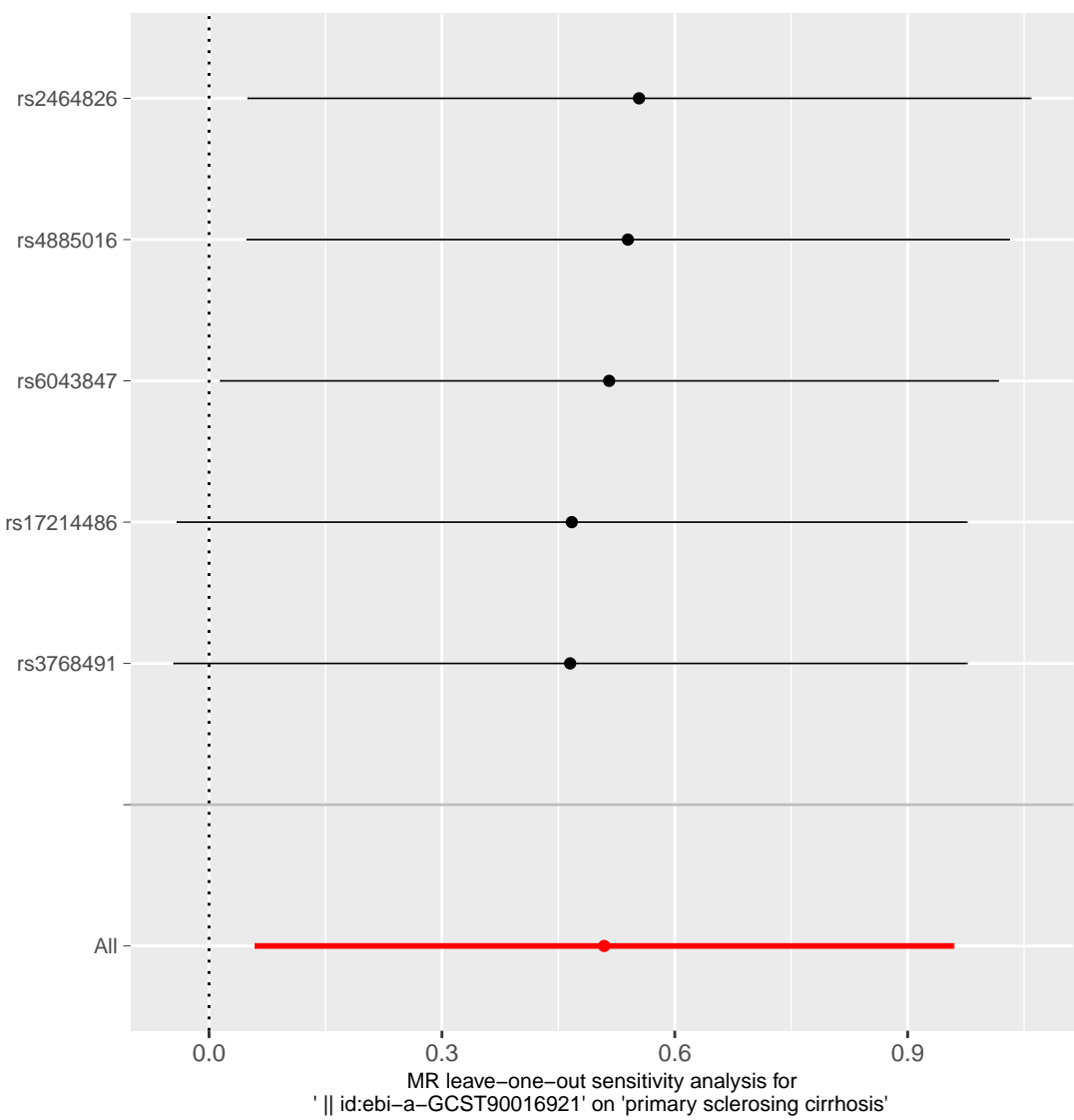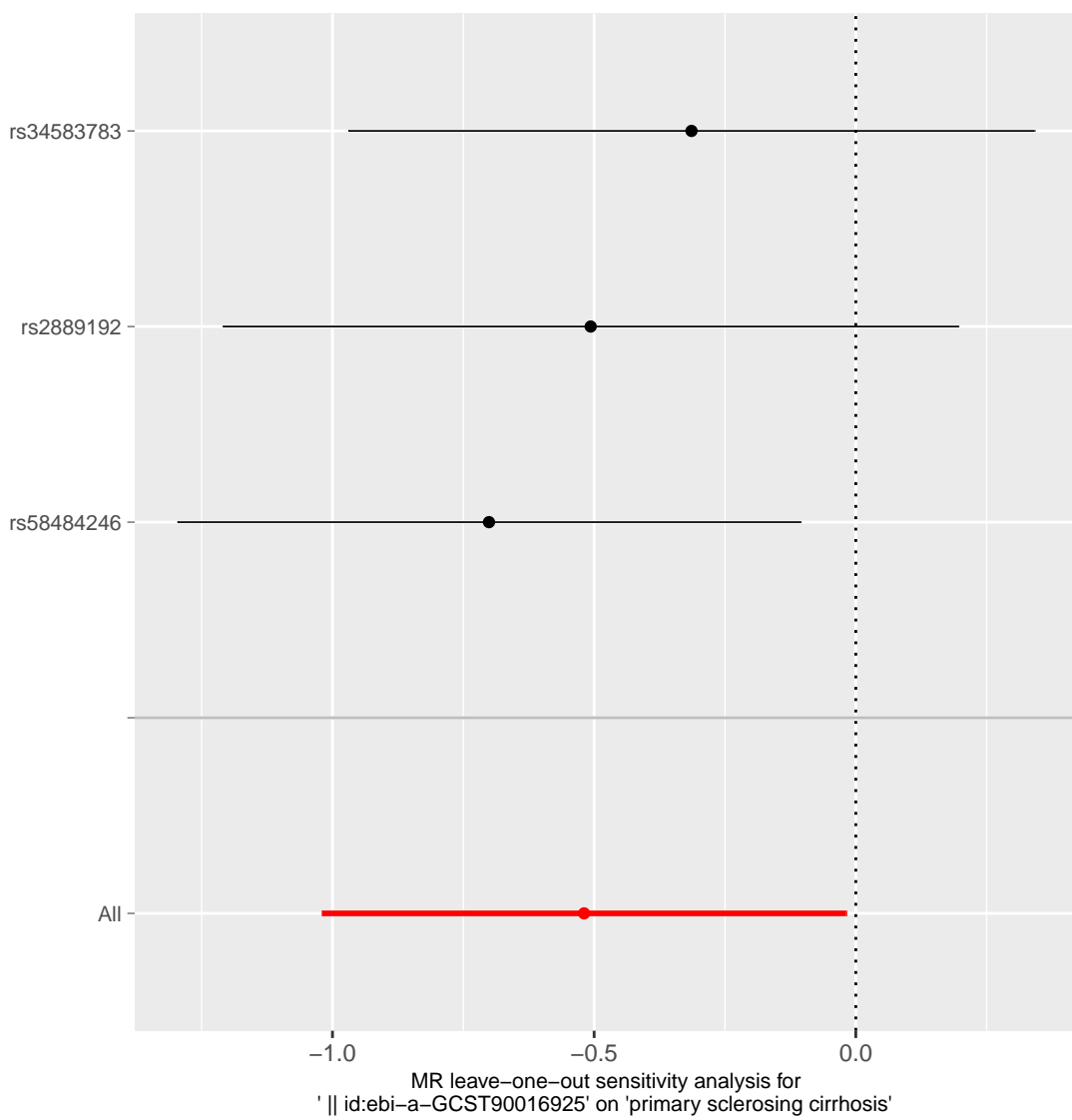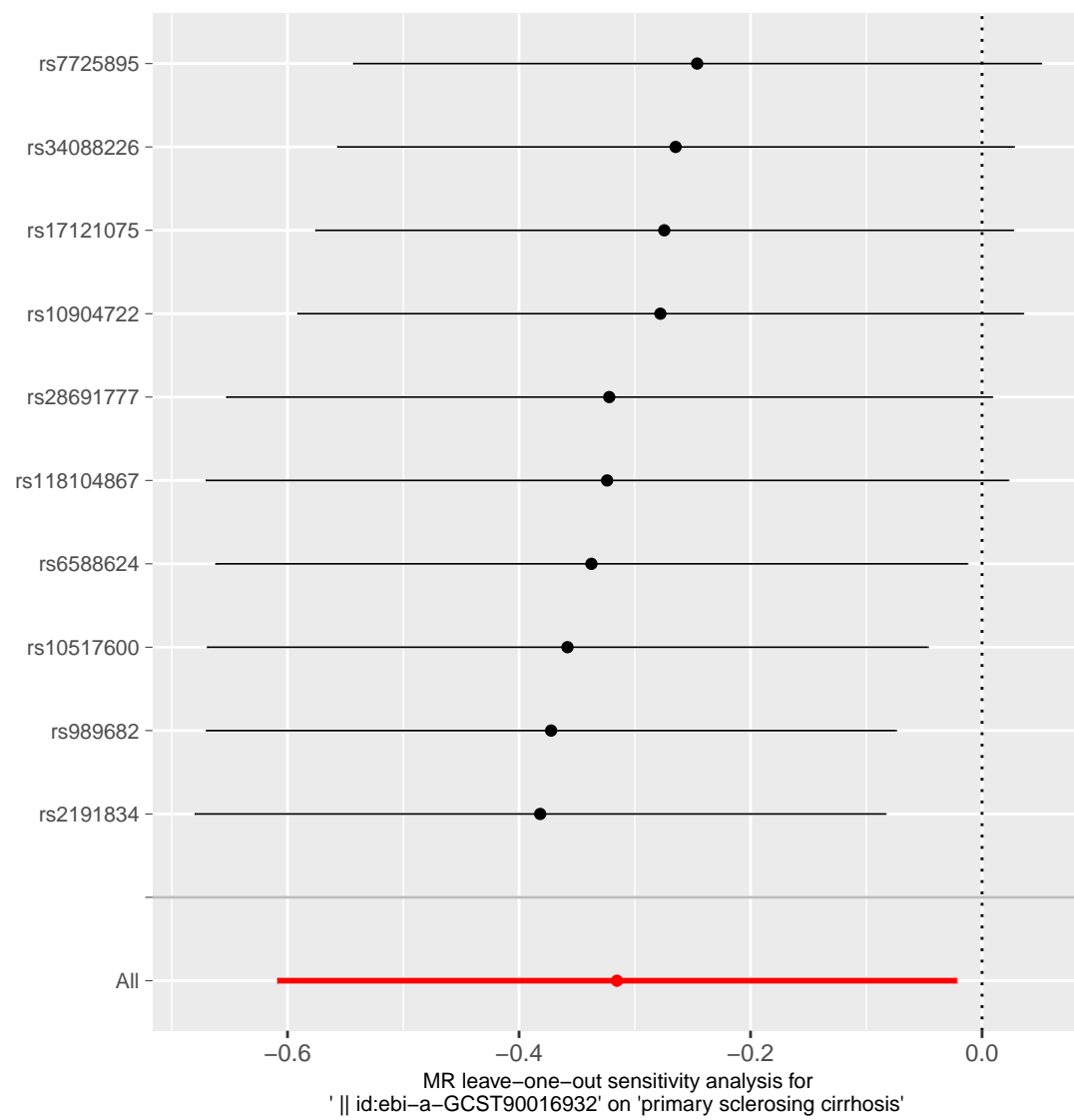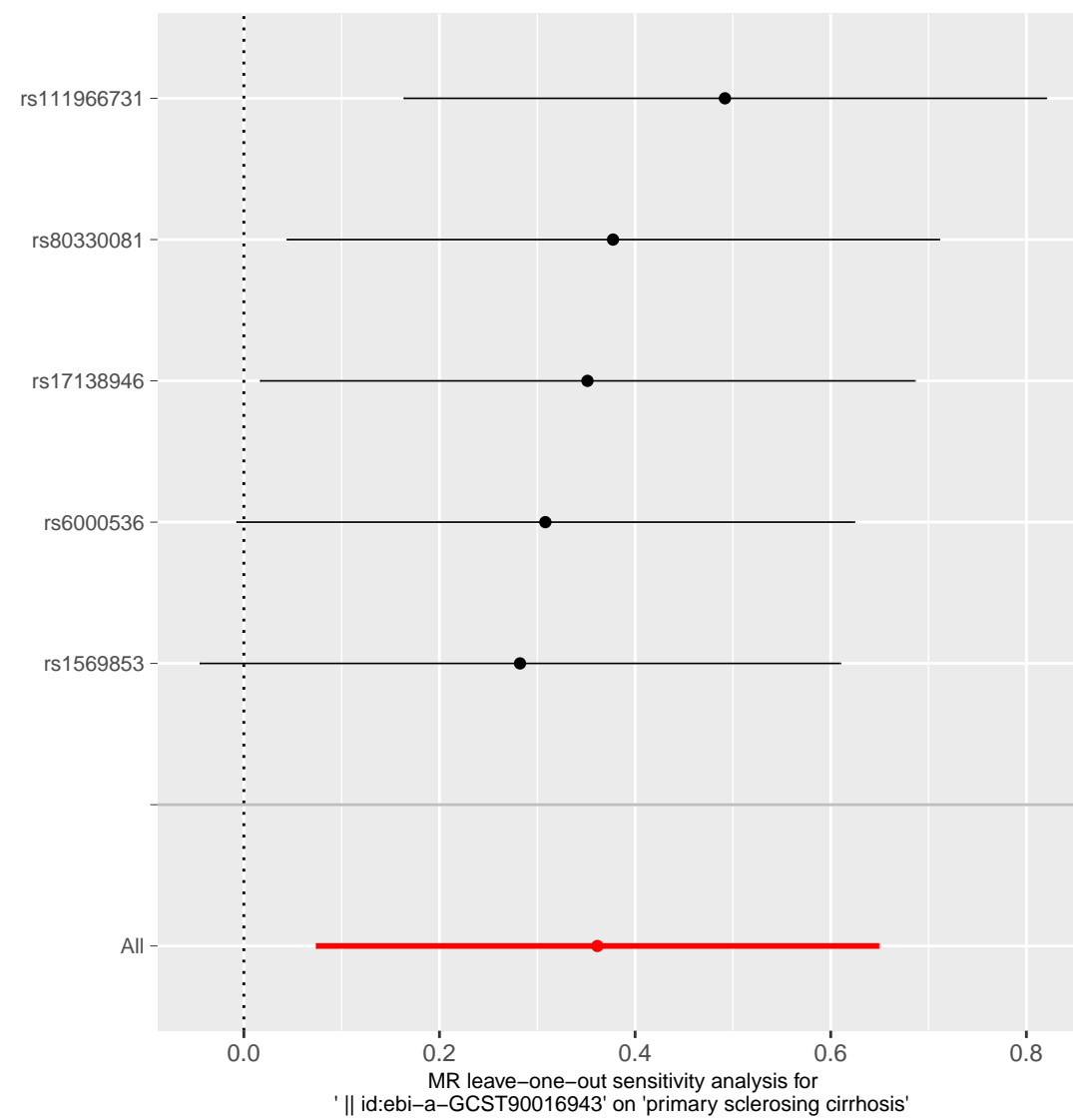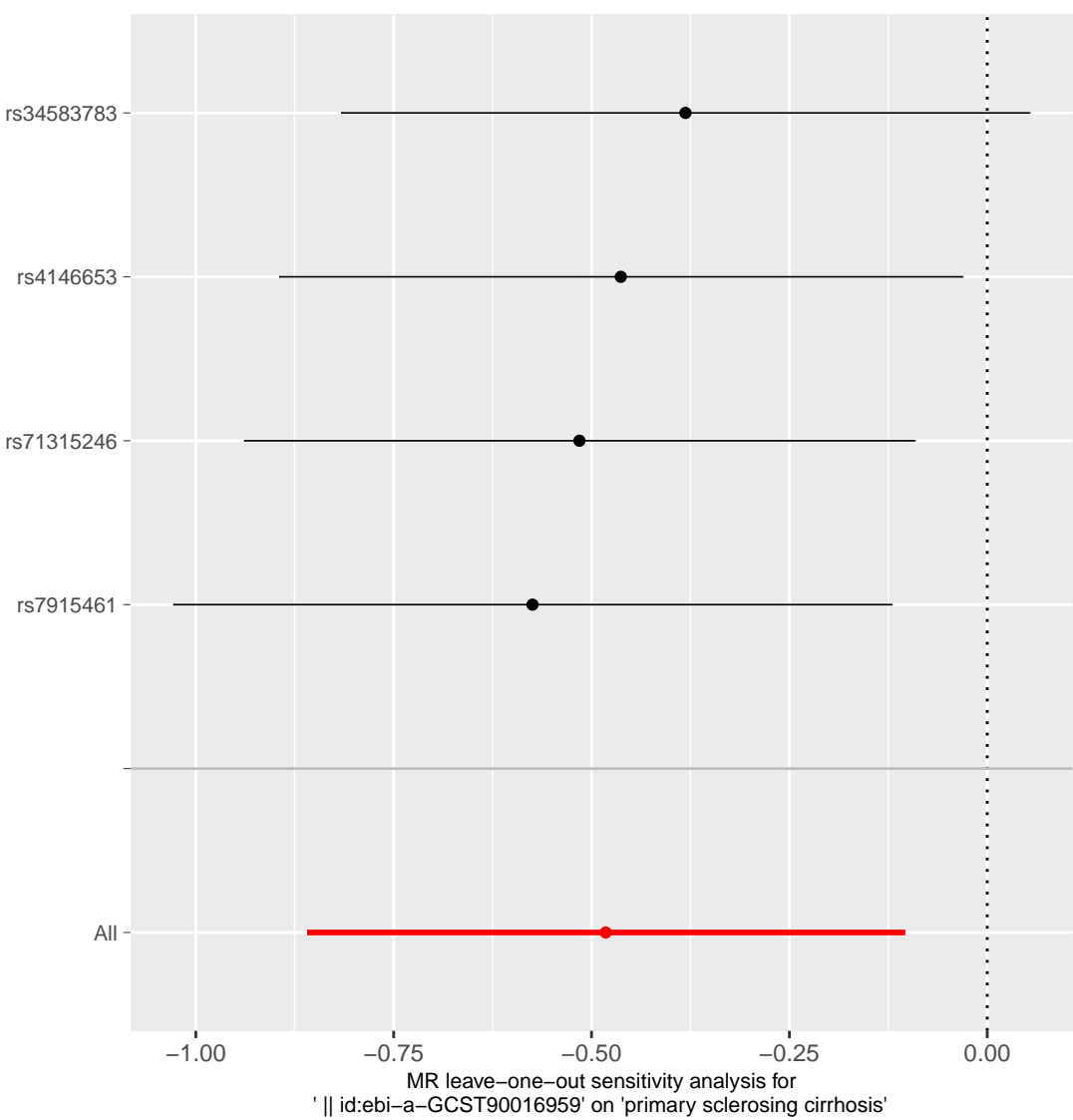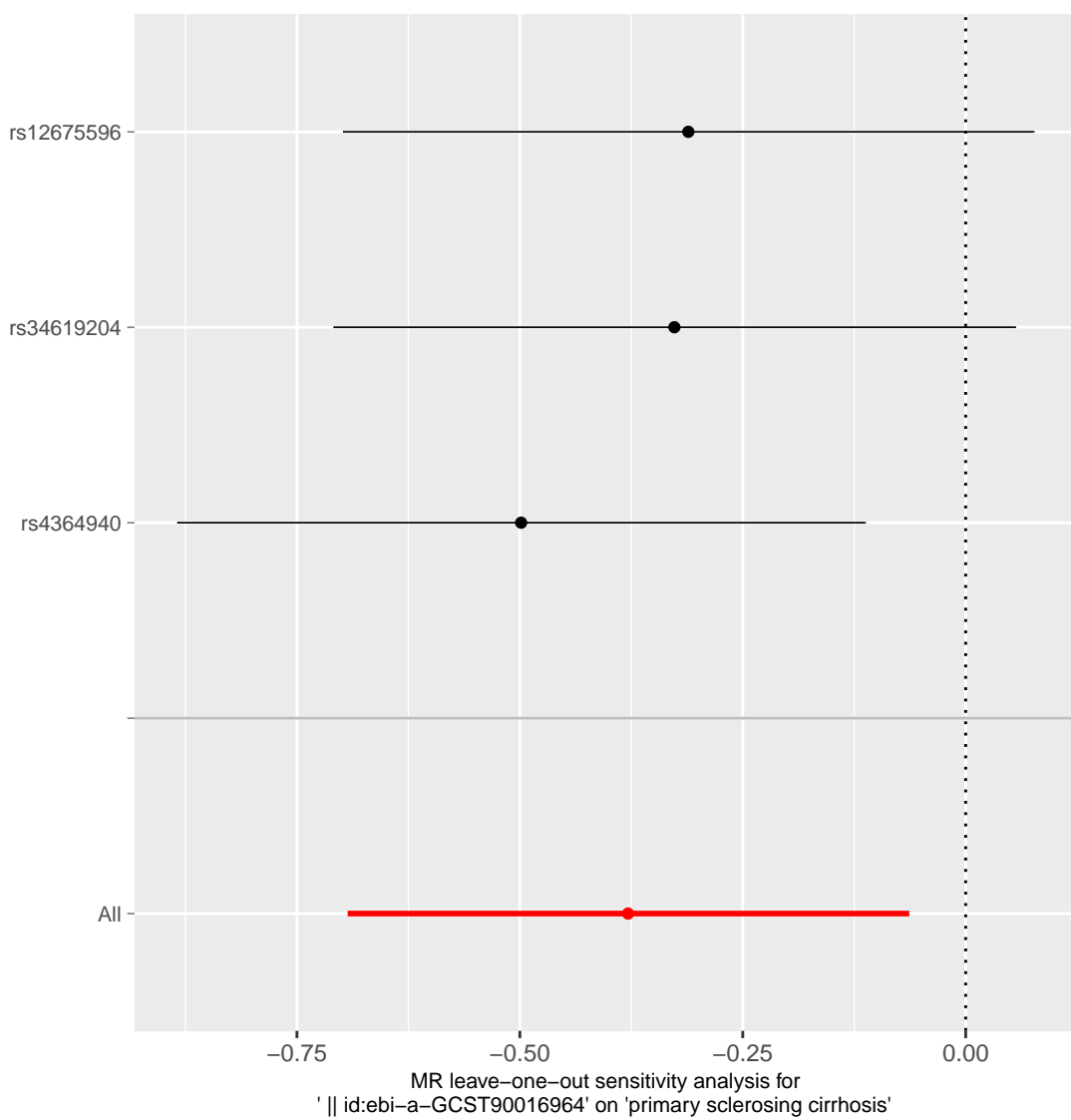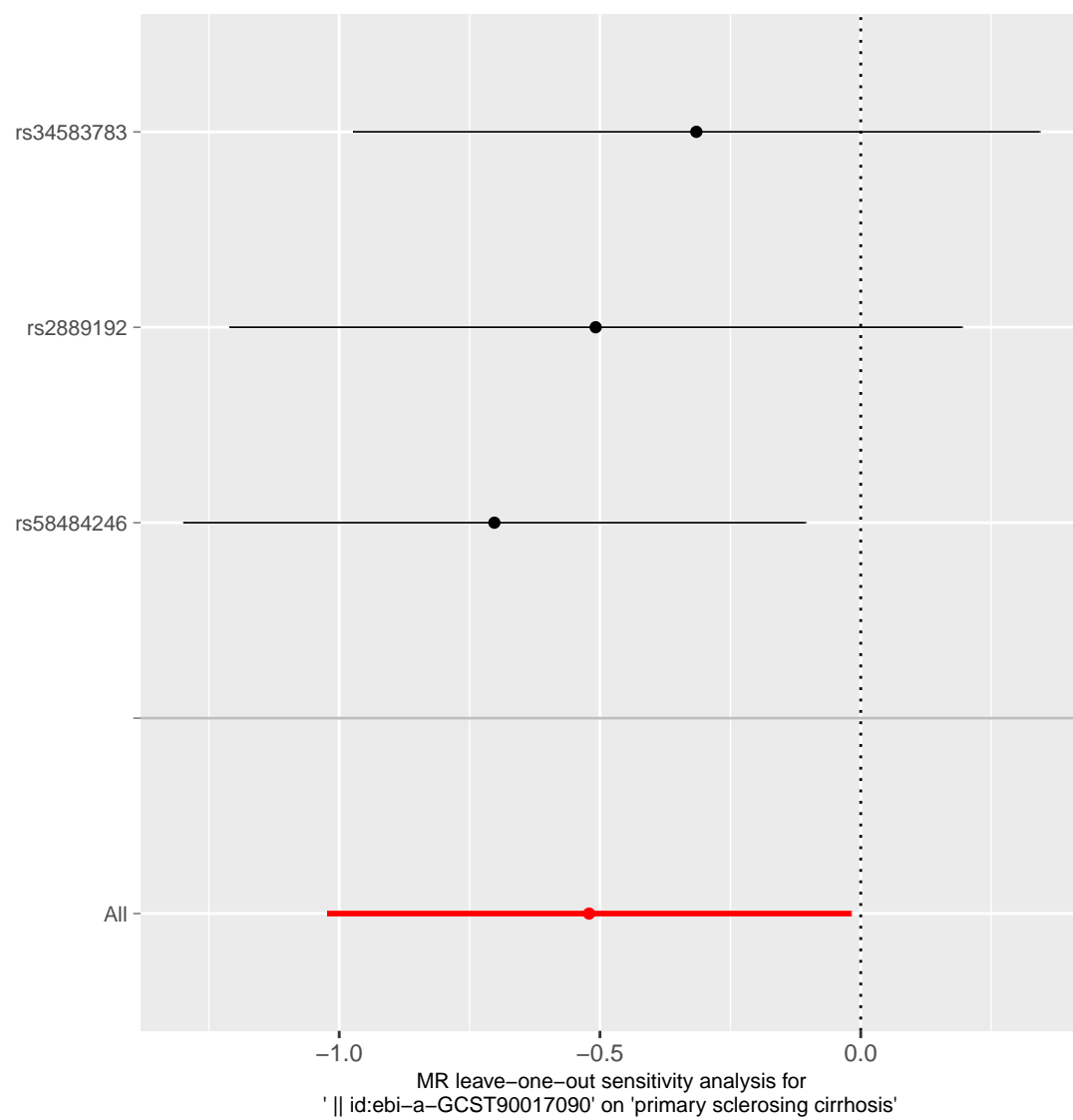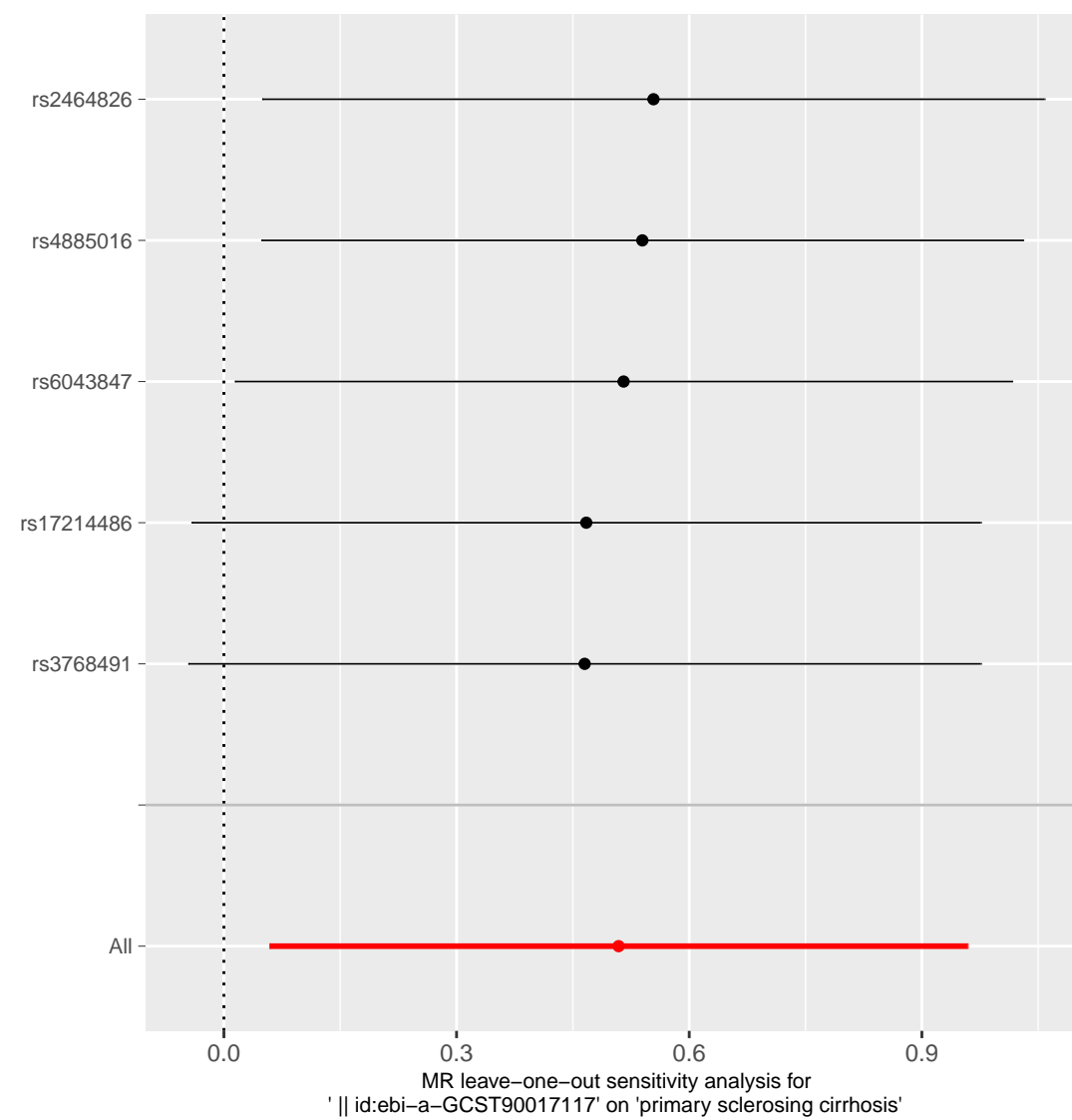

Supplement: Supplementary file 1 — Additional file 1: Supplementary Figure S1-3. Results of sensitivity analyses displayed in scatter plots for AIH (S1), PBC (S2) and PSC (S3). Supplementary Figure S4-6. Results of leave-one-out analyses, evaluating the influence of individual SNPs on the associations for AIH (S4), PBC (S5) and PSC (S6). Supplementary Table S1. Instrumental Variables for each baterial triats. Supplementary Table S2. Positive results of MR analyses. Supplementary Table S3. All MR results for 194 traits. Supplementary Table S4. Results of sensitivity analyses for IVW positive MR analyses. Supplementary Table S5. Results of MR-PRESSO. [file 12920_2023_1670_MOESM1_ESM.zip › Supplemental Materials/figs/S6 leaveoutplot_psc.pdf]
